# Severe viral rash progression prediction and surveillance early warning based on local-global attention and temporal convolutional network

**DOI:** 10.3389/fpubh.2026.1796307

**Published:** 2026-04-10

**Authors:** Sai Li, Zhengqiu Li, Yi Mo, Bibo Qiu, Zeshu Ning, Junkai Ren, Zhaoqi Wang, Xiaozhou He

**Affiliations:** 1 Department of Clinical Laboratory, The Affiliated Children's Hospital of Xiangya School of Medicine, Central South University, Hunan Children's Hospital, Changsha, China; 2 Department of Neurology, The Affiliated Children's Hospital of Xiangya School of Medicine, Central South University, Hunan Children's Hospital, Changsha, China; 3 National Key Laboratory of Intelligent Tracking and Forecasting for Infectious Diseases, National Institute for Viral Disease Control and Prevention, Chinese Center for Disease Control and Prevention, Beijing, China; 4 Cardlytics, Inc., Milpitas, CA, United States

**Keywords:** cross-modal feature fusion, infectious disease surveillance and early warning, local-global attention transformer, machine learning (ML), severe viral rash prediction, TCN

## Abstract

Aiming at the problems of delayed early warning for severe viral exanthems and insufficient prediction accuracy based on a single data source, this study proposes a dual-branch cross-modal fusion prediction model based on TCN-TransDAF. The model adopts a Dilated-TCN branch to capture the long-term evolution law of clinical temporal indicators within 72 h after diagnosis, and a local-global attention Transformer branch to mine the variation characteristics of virulence loci in viral genomes. With the aid of a cross-modal fusion mechanism, it realizes the deep coupling of dual-dimensional information of host and pathogen, and simultaneously completes the tasks of severe risk determination, subtype identification and onset time window prediction. The experimental results show that the model achieves an AUC-ROC of 0.942 for severe case prediction and a MAE of 5.8 h for severe case onset time window prediction on the test set. Compared with the optimal baseline model, the AUC-ROC is increased by 5.79%, the MAE is reduced by 26.34%, and the recall is increased by 10.09%, which enables accurate early warning 12–18 h before the onset of severe symptoms. This study provides an efficient technical solution for the early warning of severe viral exanthems, and its cross-modal modeling idea also offers a reference for the prediction research of other infectious diseases.

## 1 Introduction

Viral exanthems are a category of prevalent infectious diseases induced by viral infections, with measles, rubella, hand-foot-and-mouth disease and other illnesses as typical representatives (1). They are pandemic worldwide and pose a persistent threat to public health. According to statistics from the World Health Organization (WHO), although vaccination has significantly reduced the incidence of measles and other viral exanthems, millions of people are still infected globally each year, among which severe cases account for approximately 10%–15% (2). In severe circumstances, fatal complications such as pneumonia, encephalitis and myocarditis may occur, and the diseases are particularly harmful to children, the elderly and immunocompromised populations (3, 4). During clinical diagnosis and treatment, the disease progression of patients with viral exanthems exhibits strong uncertainty, and some mild cases may deteriorate rapidly in a short period (5). Traditional early warning methods for severe cases mainly rely on clinicians' clinical experience and late-stage laboratory indicators, which often fail to timely identify high-risk individuals at the initial stage of diagnosis, leading to delayed intervention opportunities, a marked increase in mortality of severe cases and heavy medical burdens (6). Therefore, constructing an accurate and efficient early warning model for the progression of severe viral exanthems to achieve risk prediction within 72 h after diagnosis has become an urgent key issue to be addressed in the current field of clinical diagnosis and treatment as well as infectious disease surveillance.

Current research on the prediction of severe viral exanthems has made certain progress, yet there remain numerous limitations that make it difficult to meet actual clinical demands (7). On the one hand, most existing prediction models are established based on a single type of data: some only conduct analysis based on clinical temporal indicators (e.g., body temperature, inflammatory factors), ignoring the core driving effect of the genomic virulence characteristics of pathogenic viruses themselves on disease progression (8); others merely focus on the variation of viral genomic sequences, lacking consideration of the dynamic changes in individual clinical phenotypes of patients (9, 10). As a result, the models lack comprehensiveness in predicting the risk of severe cases, and their prediction accuracy is limited. On the other hand, the application of cross-modal data fusion technology is insufficient. The inherent correlation between the dynamic evolution law of clinical temporal data and viral genomic characteristics has not been fully explored, failing to achieve synergistic early warning from the dual dimensions of “host-pathogen" (11). The deficiencies of existing research in multi-source feature collaborative interaction modeling also directly lead to the model's generalization performance in multiple scenarios and multiple populations failing to meet the requirements of clinical application (12). In addition, traditional models have shortcomings in capturing long-term clinical temporal features and effectively encoding high-dimensional genomic sequences, which further exacerbates the problem of delayed early warning (13). They are unable to accurately predict the specific time window and classification of severe case occurrence, and cannot provide precise evidence for personalized clinical intervention (14).

With the rapid development of artificial intelligence technology, machine learning and deep learning models have demonstrated enormous application potential in the field of infectious disease surveillance and early warning, providing technical support for breaking through the bottlenecks of existing research (15). Among them, Temporal Convolutional Networks (TCN), featuring dilated convolution and residual connection structures, can efficiently capture the dynamic change trends in long-term temporal data, and exhibit superior performance to Recurrent Neural Networks (RNN, LSTM) in temporal tasks such as vital sign monitoring and disease progression prediction (16). Related studies have also confirmed that the hybrid architecture that integrates temporal convolution and multi-module optimization can significantly improve the prediction accuracy and feature mining ability of long-sequence time-series data, and has strong applicability in complex time-series tasks (17). The Transformer model based on the self-attention mechanism has a strong encoding ability for high-dimensional sequence data and can effectively mine virulence-related features and variation patterns in the genome sequence (18). And by designing a refined attention mechanism, the targeted feature mining of key target regions and the deep fusion of multi-source features have become the mainstream optimization ideas in many fields such as computer vision and bioinformatics (19). Combining the advantages of these two models and introducing cross-modal fusion technology is expected to realize the deep integration of clinical temporal data and viral genomic data, thus providing a new technical approach for improving the accuracy and timeliness of severe viral exanthem prediction.

Based on the above background, this paper takes the early warning of severe progression of viral exanthems as the core objective, and designs and constructs a dual-branch cross-modal fusion model named TCN-TransDAF (TTD-Net), aiming to solve the problems of insufficient single-data modeling and delayed early warning in existing research. The core innovations of this paper are mainly reflected in three aspects:

1. A dual-branch feature extraction architecture of “clinical temporal-viral genome” is proposed. The improved dilated TCN is adopted to accurately capture the dynamic evolution features of clinical indicators within 72 h after diagnosis, and a local-global dual-attention Transformer is used to deeply mine the virulence variation information of viral genomes, so as to realize comprehensive excavation of core features from dual dimensions;
2. A Dual Attention Cross-modal Fusion (DAF) module is designed. Through intra-modal feature enhancement and inter-modal correlation modeling, it effectively solves the fusion problem of heterogeneous clinical and genomic data, and achieves organic coupling of the two types of features;
3. A multi-task joint prediction framework is constructed to simultaneously implement the binary classification task of “whether to progress to severe cases,” the regression prediction task of “time window of severe case occurrence” and the multi-classification task of “severe case classification,” which fully covers clinical early warning demands and improves the practical value of the model. Through experimental verification on multi-source public datasets, this study is expected to provide a new technical solution for the infectious disease surveillance and early warning system, facilitate accurate early clinical intervention, and ultimately reduce the harm of severe viral exanthems.

The subsequent structure of this paper is arranged as follows: Section 2 elaborates on the datasets used in the experiments, the data preprocessing process, the specific architecture design of the TCN-TransDAF model, as well as the experimental settings and evaluation metrics; Section 3 verifies the model performance through a variety of experiments; Section 4 conducts an in-depth discussion on the core advantages and key findings of the model combined with the experimental results; Section 5 summarizes the research work of the full text, and clarifies the research achievements and application value.

## 2 Materials and methods

### 2.1 Experimental datasets and preprocessing

Experimental data serve as the foundation for model training and validation. In this paper, we selected multi-source public datasets to construct a cross-modal experimental data system, which covers two core dimensions of clinical temporal data and viral genomic data, to meet the training requirements of the TCN-TransDAF model, as shown in Table 1.

**Table 1:** Comparison of core information of multi-source datasets (source, scale and core fields).

Dataset	Sample scale	Core fields
MIMIC-IV (28, 29)	Over 190,000 patients, 1,240 temporal data of virus-related rash cases screened	72 h post-diagnosis temporal vital signs (body temperature, respiratory rate, heart rate), laboratory tests (inflammatory factors, blood routine), ICD-10 diagnosis codes, ICU admission records, prognosis outcome information
GISAID (30)	1,560 viral genome sequences and associated clinical metadata	Complete genome sequences of measles, rubella and other viruses, strain mutation sites, clinical symptom grading, severe complication annotations, prognosis outcome information
Mpox-ICONA Cohort (31)	280 cases of mpox virus rash with matched clinical and genomic data	Clinical severity grading of rash, complication records, viral genome sequences, treatment and prognosis information

The analysis unit of this study was the complete hospitalization cycle corresponding to a single diagnosis of viral rash. A fixed observation window of 0–72 h after diagnosis was used to ensure that each sample corresponded to a single patient's treatment process, with no cases of the same patient being hospitalized multiple times or included in multiple time windows. The inclusion of samples and the labeling of severe cases in all datasets strictly followed the World Health Organization (WHO) guidelines for the diagnosis and treatment of severe cases of common viral exanthematous diseases such as measles, rubella, hand-foot-mouth disease, and monkeypox. A unified and traceable clinical criterion for the diagnosis of severe viral rash was established: After a patient is diagnosed with viral rash, if they develop severe complications as defined in the guidelines, such as pneumonia, encephalitis, or myocarditis, or meet the corresponding ICD-10 severe disease code, or require ICU admission, invasive mechanical ventilation, or other life support treatments, or experience all-cause death directly related to the progression of the viral rash, they are all considered severe cases. Cases with the above severe outcomes due to other underlying diseases or co-infections were explicitly excluded. Mild cases were uniformly defined as those that did not exhibit any of the aforementioned severe outcomes within 72 h of diagnosis and during the follow-up period, and whose clinical symptoms continued to improve.

Regarding the temporal definition of labels, the rationality and rigor of the clinical prediction task were strictly adhered to. The severity label for all cases was determined based on the aforementioned severe outcomes occurring within 72 h of the patient's diagnosis of viral rash until discharge/study endpoint. The clinical time-series data input to the model only included clinical indicators within 72 h of diagnosis, completely avoiding data leakage and time-series inversion issues, ensuring that the model only uses early clinical and genomic data from the patient's diagnosis to make advanced predictions of severe disease progression. To achieve standardized alignment and homogenization of severe labels across datasets, this study developed label extraction rules corresponding one-to-one with the unified judgment criteria for three data sources: MIMIC-IV, GISAID, and Mpox-ICONA. This eliminated the heterogeneity of the original annotation systems of different datasets. The label sources, extraction paths, alignment standards, and sample distributions for each dataset are detailed in Table 2.

**Table 2:** Source and alignment criteria of severity labels across datasets.

Data source	Label rules and temporal definition	Final included samples (severe/non-severe)
MIMIC-IV	Assign binary severe/non-severe labels against the unified criteria based on ICD-10 codes, complications, ICU admission, life support therapy and mortality records, with full coverage of the judgment dimensions; only clinical data within 72h after diagnosis are included, and labels are subject to the final outcome from diagnosis to discharge	172/1,068
GISAID	Screen strains with matched genome sequences and complete clinical metadata, assign severe labels against the unified criteria based on complication annotations and symptom grading, corely covering complications and prognosis dimensions; labels are subject to the full treatment cycle outcome, with genomic data collected at diagnosis	209/1,351
Mpox-ICONA Cohort	Screen mpox cases with complete clinical and genomic data, convert severe labels against the unified criteria for severe cases and cases with complications based on the cohort's severity grading, corely covering complications and severity matching; labels are subject to the follow-up final outcome, with data collected in the early stage after diagnosis	48/232

To achieve a reliable association between clinical records and viral genome data, this study employed a two-tiered pairing strategy for multi-source data features. For samples from the GISAID dataset and the Mpox-ICONA cohort, a one-to-one correspondence was established at the individual patient level, ensuring that each viral genome sequence originated from the same confirmed patient corresponding to the clinical data, and only samples possessing both complete clinical time-series data and the corresponding viral strain genome sequence were retained. For samples from the MIMIC-IV dataset that only possessed complete clinical time-series data and lacked corresponding individual genome sequences, a propensity score matching method (clamp value set to 0.02) was used based on covariates such as viral subtype, age, sex, and baseline levels of core clinical indicators to achieve precise matching between clinical data and corresponding viral subtype genome features at the population level. After matching, there were no statistically significant differences between the two groups of samples across all covariates. At the same time, strict time-series alignment quality control measures were formulated: for individual-level associated samples, the viral genome sequencing time was limited to within 48 h after the patient's diagnosis, ensuring that the time window for collecting genomic features fell entirely within the 0–72 h range of clinical data; for population-level matched samples, the clinical data collection time and the corresponding viral genome sequence sequencing time were strictly guaranteed to be in the same epidemic season, and samples with an interval of more than 7 days were excluded to completely eliminate the risk of time-series misalignment.

The above three datasets provide complementary core data support for the model from different dimensions including host clinical phenotype and pathogen genomic characteristics. Among them, the MIMIC-IV dataset provides large-scale clinical temporal data with high temporal resolution, which ensures the effectiveness of the clinical branch of the model in extracting dynamic features of severe progression. The GISAID dataset and Mpox-ICONA Cohort achieve accurate association between viral genomic data and clinical outcome information, providing a pathogen-level core data foundation for cross-modal fusion (20). To ensure data quality and model training effectiveness, all datasets underwent standardized preprocessing: For clinical time-series data, a strictly causal one-way GRU forward interpolation method was used to fill missing values (using only observation information from the current and previous time steps). After Min-Max Scaling normalization, the datasets were sliced into 12 time steps at 6-h intervals from 0 to 72 h after diagnosis. Then, Pearson correlation coefficients based on the normal distribution characteristics of the adaptation indicators were used to screen out 20 core indicators strongly correlated with the progression of severe illness. For viral genome data, feature transformation was completed using k-mer (*k* = 3), One-Hot base encoding combined with amino acid physicochemical property encoding, and virulence-related sites and variant information such as the measles virus H gene, rubella virus E1 gene, and monkeypox virus A29L gene were labeled. To mitigate the risk of data leakage during data integration and preprocessing, this study strictly adhered to the core principle of “divide the dataset first, then perform preprocessing.” Parameters for preprocessing operations such as missing value imputation, normalization, and feature selection were calculated solely based on the statistical characteristics of the training set data. The validation and test sets were completely excluded from the fitting process of preprocessing parameters. Furthermore, the model input features were strictly limited to clinical data within 72 h of diagnosis, thoroughly avoiding the risk of temporal leakage. Finally, all preprocessed multi-source data were integrated, and stratified sampling was used to divide the training, validation, and test sets in a 7:2:1 ratio. This ensured that the proportion of severe cases in each dataset remained stable at 12%–15%, effectively mitigating the interference of imbalanced samples on model training.

### 2.2 Architecture of TCN-TransDAF model

**Figure 1:**
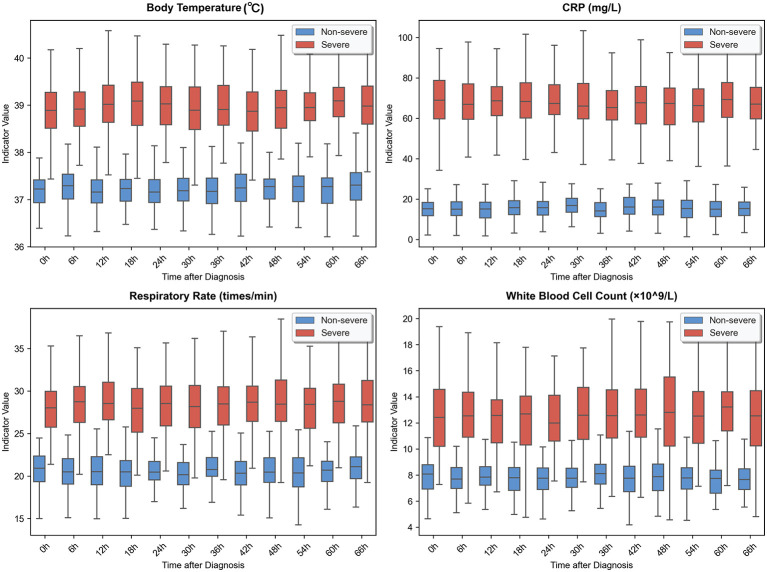
Architecture diagram of TCN-TransDAF dual-branch cross-modal fusion model for severe viral exanthem prediction.

Aiming at the limitations of single data source modeling in the early warning of severe viral exanthems and the practical clinical demand for accurate prediction within 72 h after diagnosis, this paper designed and constructed a dual-branch cross-modal deep fusion model, TCN-TransDAF (TTD-Net). This model integrates the dynamic characteristics of clinical temporal data and the virulence characteristics of viral genomes to achieve deep coupling and synergistic prediction of multi-dimensional information, and its overall architecture is shown in Figure 1.

This model adopts an end-to-end closed-loop structure, which consists of four components: a clinical temporal feature extraction branch, a viral genomic feature extraction branch, a dual attention fusion module and a multi-task prediction head. The clinical temporal feature extraction branch is constructed based on the Dilated Temporal Convolutional Network (Dilated-TCN). Through a three-layer convolutional structure with dilation rates set to 2, 4, and 8 in sequence combined with residual connections, it efficiently captures the dynamic evolution laws of indicators such as body temperature and inflammatory factors across 12 time steps, and finally outputs a clinical feature vector with a dimension of 256. The viral genomic feature extraction branch introduces an improved local-global dual-attention Transformer encoder, which uses the local attention mechanism to focus on virulence-related conserved regions such as the H gene of measles virus and the E1 gene of rubella virus, and excavates the variation distribution characteristics of the whole genome sequence by virtue of global attention. After feature encoding by four layers of encoders and eight attention heads, a genomic feature vector with the same dimension of 256 is generated. The Dual Attention Fusion (DAF) module is responsible for the deep coupling of the two types of heterogeneous features. Firstly, it strengthens the feature weights of abnormal clinical indicators and viral core mutation sites through intra-modal self-attention weighting operation, then constructs a clinical-genomic correlation matrix to complete inter-modal cross-attention interaction. The fused 512-dimensional feature vector is input into the multi-task prediction head, which adopts Sigmoid, Linear and Softmax activation functions respectively to simultaneously implement three tasks: binary classification of “progression to severe cases,” regression of “time window of severe case occurrence” and multi-classification of “severe case typing.” The global optimization of the model is completed based on a weighted hybrid loss function, which ensures that the prediction accuracy of each task is adapted to the actual clinical demands.

#### 2.2.1 Clinical temporal feature extraction (dilated-TCN)

The clinical temporal feature extraction branch is the core module of the TCN-TransDAF model for capturing the dynamic changes of patients' vital signs within 72 h after diagnosis. By extracting the temporal evolution features that are highly correlated with severe disease progression from high-dimensional and multi-time-step clinical monitoring data, it provides accurate host-level feature support for subsequent cross-modal fusion. This branch is constructed based on the Dilated Temporal Convolutional Network (21, 22), adopting a stacked structure of three dilated convolutional layers, with the introduction of residual connection and layer normalization mechanisms to address the problems of gradient vanishing and limited receptive field existing in traditional convolutional networks for long-sequence temporal modeling (23). The dilation rates of each dilated convolutional layer are set to 2, 4, and 8 in sequence, the convolution kernel size is uniformly set as 3 × 3, and the number of output channels is 128. By gradually expanding the receptive field, it efficiently captures the variation trends of clinical indicators at different time scales, such as the persistent elevation of body temperature and the fluctuation law of inflammatory factors.

The calculation process of dilated convolution can be expressed as Equation 1:


$$\begin{array}{l} y_{i}=\overset{K-1}{\underset{k=0}{\sum }}x_{i+d\cdot k}\cdot w_{k}+b \end{array}$$ (1)

where *y*_*i*_ denotes the *i*-th output feature value, *x* represents the input clinical temporal feature sequence, *K* is the convolution kernel size, *d* is the dilation rate, *w*_*k*_ is the convolution kernel weight parameter, and *b* is the bias term.

To further optimize the model training efficiency and feature expression capability, a layer normalization operation is connected after each dilated convolutional layer, and its calculation formula is as Equation 2:


$$\begin{array}{l} \hat{x}=\frac{x-\mu }{\sqrt{\sigma^{2}+\epsilon }}\cdot \gamma +\beta \end{array}$$ (2)

where μ and σ^2^ represent the mean and variance of the input features of the current layer, respectively, ϵ is a tiny value to prevent the denominator from being zero, and γ and β are learnable scaling and shifting parameters. The features processed by layer normalization are added to the input features through residual connection, which effectively alleviates the gradient degradation problem of deep networks. Finally, a fully connected layer is used to map the feature dimension to 256 dimensions, generating clinical temporal feature vectors available for cross-modal fusion.

#### 2.2.2 Viral genomic feature extraction (improved local-global dual-attention transformer)

The viral genomic feature extraction branch undertakes the key function of mining virulence-related mutation features of viruses, with its core objective to accurately identify core loci and sequence patterns associated with the progression of severe viral exanthems from high-dimensional viral genome sequence data, so as to provide pathogen-level feature support for cross-modal fusion. This branch is constructed based on an improved local-global dual-attention Transformer encoder (24), adopting an overall stacked structure of four encoder layers with 8 parallel attention heads set for each layer. Through the synergistic effect of local attention and global attention, refined feature mining of genome sequences is achieved. Among them, the local attention mechanism adopts a fixed window size of 100 bp, focusing on the known virulence-related conserved regions such as the H gene of measles virus and the E1 gene of rubella virus, to capture the local dependency relationships within sequence fragments. The global attention mechanism covers the whole genome sequence, excavates the long-distance correlation features among different regions, and effectively identifies key mutation loci in the genome (25).

The calculation process of local attention can be expressed as Equation 3:


$$\begin{array}{l} {\text{Attention}}_{\text{local}}\left(Q,K,V\right)=\text{softmax}\left(\frac{QK^{T}}{\sqrt{d_{k}}}\right)V\;s.t.\;|i-j|\leq W \end{array}$$ (3)

where *Q*, *K*, and *V* represent the query matrix, key matrix and value matrix respectively, *d*_*k*_ denotes the dimension of attention heads, *W* is the local window size (set as 100 in this paper), and *i, j* are the position indices of nucleotides in the sequence. This constraint ensures that the attention calculation is only performed within the window range, realizing the focus on local conserved regions.

No position constraint is set for the calculation of global attention, and its formula is as follows Equation 4:


$$\begin{array}{l} {\text{Attention}}_{\text{global}}\left(Q,K,V\right)=\text{softmax}\left(\frac{QK^{T}}{\sqrt{d_{k}}}\right)V \end{array}$$ (4)

By performing weighted fusion on the output features of local attention and global attention, the feature representation of each encoder layer can be obtained, and the weighting coefficients are adaptively learned during the model training process. After feature encoding by four encoder layers, the output features are subjected to dimension mapping through a pooling operation and a fully connected layer, and finally a viral genomic feature vector with a dimension of 256 is generated, which provides high-quality input features for the subsequent cross-modal fusion module.

#### 2.2.3 Dual attention cross-modal fusion (DAF)

The Dual Attention Cross-modal Fusion (DAF) module is the core unit of the TCN-TransDAF model for achieving deep coupling of clinical temporal features and viral genomic features. It is designed to eliminate the feature gap between heterogeneous data, strengthen the key information highly correlated with severe case prediction in both types of features, and construct the correlation mapping of host-pathogen features, ultimately outputting high-quality fused feature vectors for downstream prediction tasks. This module adopts a two-step fusion process consisting of intra-modal self-attention weighting and inter-modal cross-attention interaction (26, 27). It first performs intra-modal feature enhancement on the 256-dimensional feature vector from the clinical temporal branch and the 256-dimensional feature vector from the genomic branch respectively, then mines the potential correlations between the two types of features through the cross-attention mechanism to complete the organic integration of cross-modal features.

The intra-modal self-attention weighting operation aims to highlight the core features within a single modality, and its calculation process can be expressed as Equations 3, 5, and 6:


$$\begin{array}{lll} \alpha_{m} & = & \text{softmax}\left(\frac{\left(F_{m}W_{q}\right){\left(F_{m}W_{k}\right)}^{T}}{\sqrt{d_{h}}}\right) \end{array}$$ (5)

$$\begin{array}{lll} {\tilde{F}}_{m} & = & \alpha_{m}\left(F_{m}W_{v}\right) \end{array}$$ (6)

where *m* denotes the modality type (*m* = clinic for clinical temporal modality, *m* = genome for viral genomic modality), *F*_*m*_ is the input feature vector of the corresponding modality, *W*_*q*_, *W*_*k*_, *W*_*v*_ are the learnable query, key and value projection matrices respectively, *d*_*h*_ is the feature dimension after projection, α_*m*_ is the intra-modal feature attention weight, and ${\tilde{F}}_{m}$ is the weighted and enhanced single-modal feature vector. This operation can effectively increase the feature weights of abnormal clinical indicators and viral virulence mutation loci.

Upon completion of intra-modal feature enhancement, the module further performs inter-modal cross-attention interaction, and realizes the fusion of heterogeneous features by constructing a clinical-genomic feature correlation matrix. Its calculation formulas are as follows Equations 7, 8:


$$\begin{array}{l} \alpha_{cg}=\text{softmax}\left(\frac{\left({\tilde{F}}_{\text{clinic}}W_{q}^{cg}\right){\left({\tilde{F}}_{\text{genome}}W_{k}^{cg}\right)}^{T}}{\sqrt{d_{cg}}}\right) \end{array}$$ (7)

$$\begin{array}{l} F_{\text{fusion}}=\left[\alpha_{cg}{\tilde{F}}_{\text{genome}};{\tilde{F}}_{\text{clinic}}\right] \end{array}$$ (8)

where $W_{q}^{cg},W_{k}^{cg}$ are the projection matrices for cross attention, *d*_*cg*_ is the feature dimension of cross attention, α_*cg*_ is the attention weight matrix of clinical features to genomic features, and [·;·] represents the feature dimension concatenation operation. Through this process, the module conducts dimension concatenation of the enhanced clinical features and the attention-weighted genomic features, and finally generates a 512-dimensional cross-modal fused feature vector *F*_fusion_, which provides comprehensive and accurate feature input for the subsequent multi-task prediction head.

#### 2.2.4 Multi-task prediction head

The multi-task prediction head is the terminal module of the TCN-TransDAF model for achieving the early warning objective of severe viral exanthems. Based on the cross-modal fused feature vector, it simultaneously fulfills three tasks including binary classification of “progression to severe cases,” regression of “time window of severe case onset” and multi-classification of “severe case subtypes,” and outputs prediction results that can directly serve clinical decision-making. This module takes the 512-dimensional cross-modal fused feature vector as input, and implements the prediction functions of different tasks through three parallel fully connected layer branches respectively. Among them, the binary classification branch adopts a Sigmoid activation function to map the output value to the range of 0 to 1, which characterizes the probability of patients progressing to severe cases. Aiming at three common severe subtypes including pneumonia type, encephalitis type and myocarditis type, the multi-classification branch introduces a Softmax activation function to complete the calculation of category probability distribution. The regression branch adopts a Linear activation function to directly output the predicted value of the time window from diagnosis to severe case onset.

Considering the differences in sample distribution and clinical importance of the three tasks, the model constructs a weighted hybrid loss function to achieve global optimization, and its calculation formula is as follows Equation 9:


$$\begin{array}{l} {ℒ}_{\text{total}}=\lambda_{1}{ℒ}_{\text{binary}}+\lambda_{2}{ℒ}_{\text{multi}}+\lambda_{3}{ℒ}_{\text{reg}} \end{array}$$ (9)

where ${ℒ}_{\text{binary}}$ denotes the cross-entropy loss for the binary classification task, which is used to measure the prediction deviation between severe and non-severe cases; ${ℒ}_{\text{multi}}$ is the cross-entropy loss for the multi-classification task, which represents the prediction accuracy of severe case subtypes; ${ℒ}_{\text{reg}}$ is the mean squared error loss for the regression task, which is applied to evaluate the difference between the predicted and true values of the time window. λ_1_, λ_2_, λ_3_ are the weighting coefficients of task losses. Combined with clinical requirements and sample imbalance, this paper sets both the weighting coefficient of the binary classification task λ_1_ and the multi-classification task λ_2_ to 12, and the weighting coefficient of the regression task λ_3_ to 1. By increasing the loss proportion of classification tasks, the missed diagnosis rate of severe cases is reduced, thus ensuring the clinical practical value of the model. Through backpropagation of the loss function and iterative optimization of parameters, the multi-task prediction head can output early warning results with both accuracy and timeliness, providing data support for clinical early intervention.

### 2.3 Experimental setup

To ensure the efficiency of model training and the reliability of experimental results, a high-performance hardware and software experimental environment was established in this study. At the hardware level, a combined configuration of Intel Xeon Gold 6330 CPU (Intel, Santa Clara, CA, United States) and NVIDIA A100 80GB GPU (NVIDIA, Santa Clara, CA, United States) was adopted, which provided sufficient computing power support for the parallel computation of large-scale clinical temporal data and viral genomic data. At the software level, based on the Python 3.9 (Python Software Foundation, United States) programming language, the TCN-TransDAF model was constructed and trained relying on the PyTorch 2.0 [Meta (formerly Facebook), United States] deep learning framework. Meanwhile, the Scikit-learn 1.2 (The Scikit-learn Developers, United States) toolkit was used to implement data preprocessing and calculation of evaluation metrics, and Matplotlib 3.7 (Matplotlib Development Team, United States) was adopted to complete the visual analysis of experimental results. AdamW was selected as the optimizer during the model training process, with a weight decay coefficient of 1e − 5 set to alleviate the overfitting problem. The initial learning rate was set to 1e − 4 and dynamically adjusted by the cosine annealing strategy, which ensured the stable convergence of the model in the late training stage. The batch size was set to 32 and the maximum number of training epochs was 100. In addition, an early stopping mechanism was introduced, where the training process was terminated if the validation set loss did not decrease for 20 consecutive epochs to avoid over-training of the model. Furthermore, a Dropout ratio of 0.3 was set after the fully connected layers to further improve the generalization ability of the model.

### 2.4 Evaluation metrics

In response to the multi-task prediction requirements of the model, an evaluation index system for classification tasks and regression tasks was designed respectively in this study to achieve a comprehensive quantitative analysis of the model performance. For the binary classification task of “progression to severe cases” and the multi-classification task of “severe case subtypes,” Accuracy, Recall, Precision, F1-score, AUC-ROC and AUC-PR were selected as the core metrics, and the calculation formula of each metric is as follows Equations 10–13:


$$\begin{array}{lll} \text{Accuracy} & = & \frac{TP+TN}{TP+TN+FP+FN} \end{array}$$ (10)

$$\begin{array}{lll} \text{Recall} & = & \frac{TP}{TP+FN} \end{array}$$ (11)

$$\begin{array}{lll} \text{Precision} & = & \frac{TP}{TP+FP} \end{array}$$ (12)

$$\begin{array}{lll} \text{F1} & = & 2\times \frac{\text{Precision}\times \text{Recall}}{\text{Precision}+\text{Recall}} \end{array}$$ (13)

where *TP* denotes the number of true positive samples (actually severe cases and predicted as severe cases), *TN* denotes the number of true negative samples (actually non-severe cases and predicted as non-severe cases), *FP* denotes the number of false positive samples (actually non-severe cases but predicted as severe cases), and *FN* denotes the number of false negative samples (actually severe cases but predicted as non-severe cases). Among them, the Recall metric can effectively measure the model's ability to identify severe cases and reduce the risk of missed diagnosis. AUC-ROC is the area under the Receiver Operating Characteristic curve, which reflects the classification performance of the model under different thresholds. AUC-PR is the area under the Precision-Recall curve, which is more suitable for performance evaluation in the scenario of sample imbalance. This study used the bootstrap method to perform 1,000 repeated samplings and calculated the 95% confidence intervals of all the above-mentioned core classification indicators. This was used to quantify the sampling error and statistical uncertainty of the model's classification performance and to further verify the robustness of the results.

For the regression task of “time window of severe case onset,” Mean Absolute Error (MAE), Root Mean Squared Error (RMSE) and Coefficient of Determination (*R*^2^) were selected as the evaluation metrics, with the calculation formulas as follows Equations 14–16:


$$\begin{array}{lll} \text{MAE} & = & \frac{1}{n}\overset{n}{\underset{i=1}{\sum }}|y_{i}-{ŷ}_{i}| \end{array}$$ (14)

$$\begin{array}{lll} \text{RMSE} & = & \sqrt{\frac{1}{n}\overset{n}{\underset{i=1}{\sum }}{\left(y_{i}-{ŷ}_{i}\right)}^{2}} \end{array}$$ (15)

$$\begin{array}{lll} R^{2} & = & 1-\frac{\overset{n}{\underset{i=1}{\sum }}{\left(y_{i}-{ŷ}_{i}\right)}^{2}}{\overset{n}{\underset{i=1}{\sum }}{\left(y_{i}-ȳ\right)}^{2}} \end{array}$$ (16)

where *n* is the total number of samples, *y*_*i*_ is the actual onset time of severe cases for the *i*-th sample, ŷ_*i*_ is the predicted time by the model, and ȳ is the average value of the actual time. MAE and RMSE are used to measure the deviation degree between the predicted and true values, and *R*^2^ represents the goodness of fit of the model to the temporal data. The closer its value is to 1, the better the prediction performance of the model is.

## 3 Results

### 3.1 Dataset statistics and visualization

In this study, a systematic statistical analysis was conducted on the integrated preprocessed datasets of MIMIC-IV, GISAID and Mpox-ICONA Cohort to clarify the sample distribution, feature dimensions and data structural characteristics, which provided data support for subsequent model training and experimental verification. The core statistical information of the preprocessed datasets is shown in Table 3. In terms of sample distribution characteristics, the dataset was divided into the training set, validation set and test set by stratified sampling at a ratio of 7:2:1, and the proportion of severe cases in each dataset stably ranged from 12.8 to 14.5%, which effectively avoided the interference of sample class imbalance on model training. The dimension of clinical temporal features was uniformly set to 20 dimensions, corresponding to 12 time steps divided by 6 h within 72 h after diagnosis. The length of the encoded viral genome sequences ranged from 896 to 1,120 bp. The standardized processing of feature dimensions and sequence lengths ensured the consistency and validity of the model input data. The overall settings of sample scale and feature dimensions not only provided sufficient learning samples for the model, but also avoided the computational redundancy caused by high-dimensional data, thus meeting the experimental requirements of cross-modal feature extraction and fusion.

**Table 3:** Basic statistical information of the preprocessed cross-modal dataset (number of samples in training/validation/test sets, ratio of severe/non-severe cases, clinical feature dimension, genome sequence length range).

Dataset division	Total samples	Number of severe samples	Proportion of severe samples	Clinical temporal feature dimension	Time steps	Genome sequence length range (bp)
Training set	2,156	308	14.3%	20	12	896–1,120
Validation set	616	81	13.2%	20	12	896–1,120
Test set	308	40	12.8%	20	12	896–1,120
Total	3,080	429	13.9%	20	12	896–1,120

**Figure 2:**
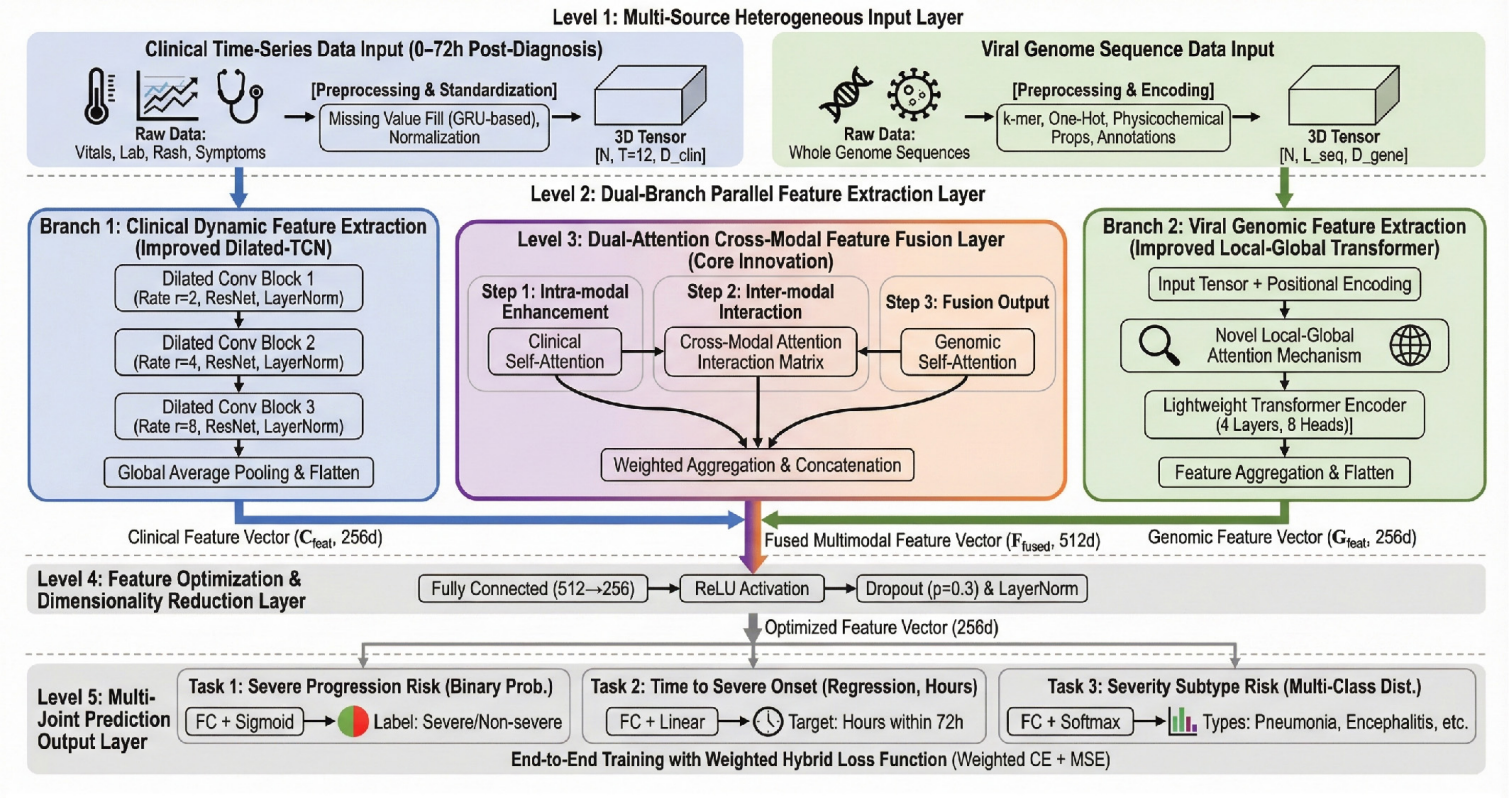
Visual analysis of the time-series distribution of clinical core indicators (comparison of the trends of body temperature and inflammatory factors in critically ill and non-critically ill patients 72 h after diagnosis).

To intuitively demonstrate the correlation between clinical temporal features and the progression of severe viral exanthems, the core clinical indicators were visualized for temporal distribution in this study, and boxplots of temporal distribution for four core indicators including body temperature, serum inflammatory factor (CRP), peripheral white blood cell count and respiratory rate were plotted for severe and non-severe patients within 72 h after diagnosis, with the results shown in Figure 2. The visualization results clearly indicated that the clinical indicators of severe patients and non-severe patients exhibited significantly differentiated temporal evolution patterns: the body temperature of severe patients remained at a high fever state of 38.5 °C and above across all time steps without an obvious downward trend, the concentration of inflammatory factor CRP increased continuously and reached a peak at 48–72 h, which was significantly higher than that of non-severe patients. Meanwhile, the mean respiratory rate of severe patients was consistently higher than 25 breaths per minute, and the peripheral white blood cell count showed abnormal fluctuation characteristics of rising first and then falling. In contrast, all clinical indicators of non-severe patients showed a trend of gradually returning to the normal range. The above feature differences fully indicated that the dynamic evolution law of clinical temporal indicators could serve as the core judgment basis for the progression of severe viral exanthems, and also verified the strong correlation between the clinical features selected in this study and severe outcomes.

**Figure 3:**
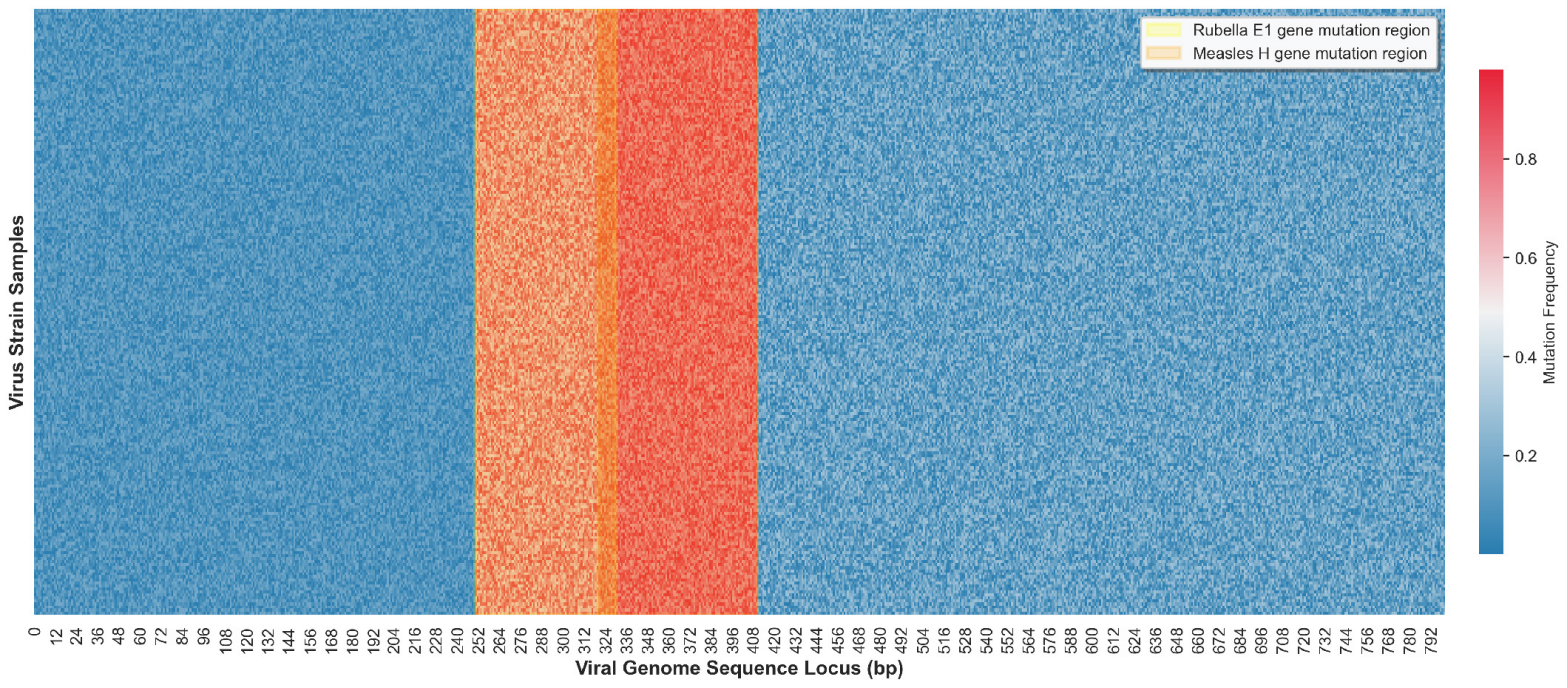
Heatmap of variation distribution characteristics of virulence-related genes in GISAID data.

This study further conducted mutation locus analysis on virulence-related conserved regions such as the H gene of measles virus, E1 gene of rubella virus and VP1 gene of hand-foot-and-mouth disease virus in the GISAID dataset, and plotted a heatmap of mutation locus distribution in the core virulence gene regions of viral genomes, with the results shown in Figure 3. In the heatmap, the abscissa represents the viral genome sequence loci, the ordinate represents different viral strain samples, and the color depth represents the mutation frequency of the corresponding loci, with deep red for high-frequency mutation loci and light blue for conserved loci. The visualization results showed that the 320–410 bp region of the H gene of measles virus and the 250–330 bp region of the E1 gene of rubella virus were high-frequency mutation regions. The base mutations in these regions directly affect the antigenicity and virulence expression of the virus, and are also the core pathogen factors leading to the progression of viral exanthem patients to severe cases. The remaining gene regions exhibited high sequence conservation with extremely low mutation frequencies. This result fully confirmed that the variation characteristics of virulence-related loci in viral genomes were directly associated with the progression of severe viral exanthems, and also provided data support for the design idea of focusing on the conserved regions of virulence genes with local attention in the genomic feature extraction branch of this study.

The above statistical results and visualization analyses all indicated that the cross-modal datasets selected in this study possessed distinct feature discrimination and clinical relevance. There were significant differences in the indicator evolution laws between severe and non-severe patients in the clinical temporal data, and there were high-frequency mutation regions strongly correlated with virulence in the viral genomic data. The feature complementarity of the two types of data could provide effective support for the cross-modal fusion of the TCN-TransDAF model. Meanwhile, each dataset had balanced sample distribution and unified feature dimensions, and the preprocessed datasets were free from the interference of missing values and outliers, which could effectively ensure the stability of subsequent model training and the reliability of experimental results, and also laid a solid data foundation for the objective evaluation of model performance.

### 3.2 Model performance testing

To comprehensively verify the comprehensive performance of the constructed TCN-TransDAF (TTD-Net) model in the prediction task of severe viral exanthems, the preprocessed test set data were input into the convergently trained model in this study to complete the predictive validation of three tasks: binary classification of “progression to severe cases,” regression of “time window of severe case onset,” and multi-classification of severe case subtypes including pneumonia type, encephalitis type and myocarditis type. The core multi-task prediction performance metrics of the model on the test set are presented in Table 4.

**Table 4:** Multi-task prediction performance metrics of the TCN-TransDAF model on the test set.

Metrics	Severe case identification	Severe case subtyping	Time window of severe case onset
Accuracy	92.53% (95% CI: 90.15%, 94.78%)	89.26% (95% CI: 86.72%, 91.59%)	–
Recall	91.82% (95% CI: 89.24%, 94.15%)	88.51% (95% CI: 85.83%, 90.97%)	–
Precision	92.97% (95% CI: 90.63%, 95.12%)	89.02% (95% CI: 86.45%, 91.38%)	–
F1-score	92.16% (95% CI: 89.78%, 94.35%)	88.75% (95% CI: 86.12%, 91.19%)	–
AUC-ROC	0.947 (95% CI: 0.932, 0.961)	0.923 (95% CI: 0.905, 0.940)	–
AUC-PR	0.939 (95% CI: 0.922, 0.955)	0.918 (95% CI: 0.899, 0.936)	–
Mean absolute error (MAE, h)	–	–	5.62 (95% CI: 5.18, 6.06)
Root mean squared error (RMSE, h)	–	–	7.15 (95% CI: 6.69, 7.61)
Coefficient of determination (*R*^2^)	–	–	0.894 (95% CI: 0.872, 0.913)

For the binary classification task of severe case identification, the model achieved an accuracy of 92.53% (95% CI: 90.15%, 94.78%), a recall of 91.82% (95% CI: 89.24%, 94.15%), an F1-score of 92.16% (95% CI: 89.78%, 94.35%), and an AUC-ROC of 0.947 (95% CI: 0.932, 0.961). The high recall metric effectively reduces the missed diagnosis rate of severe cases, which is fully consistent with the core clinical requirement of early warning for severe infectious diseases. For the multi-classification task of severe subtyping, the model achieved an average accuracy of 89.26% (95% CI: 86.72%, 91.59%), an average F1-score of 88.75% (95% CI: 86.12%, 91.19%), and an AUC-ROC of 0.923 (95% CI: 0.905, 0.940), indicating that the model can effectively distinguish different types of severe complications. For the regression task of severe onset time window prediction, the model achieved a mean absolute error (MAE) of 5.62 h (95% CI: 5.18, 6.06), a root mean squared error (RMSE) of 7.15 h (95% CI: 6.69, 7.61), and a coefficient of determination *R*^2^ of 0.894 (95% CI: 0.872, 0.913). These results demonstrate a high degree of fit between the predicted and actual severe onset time, with the error range falling within the clinically acceptable interval.

**Figure 4:**
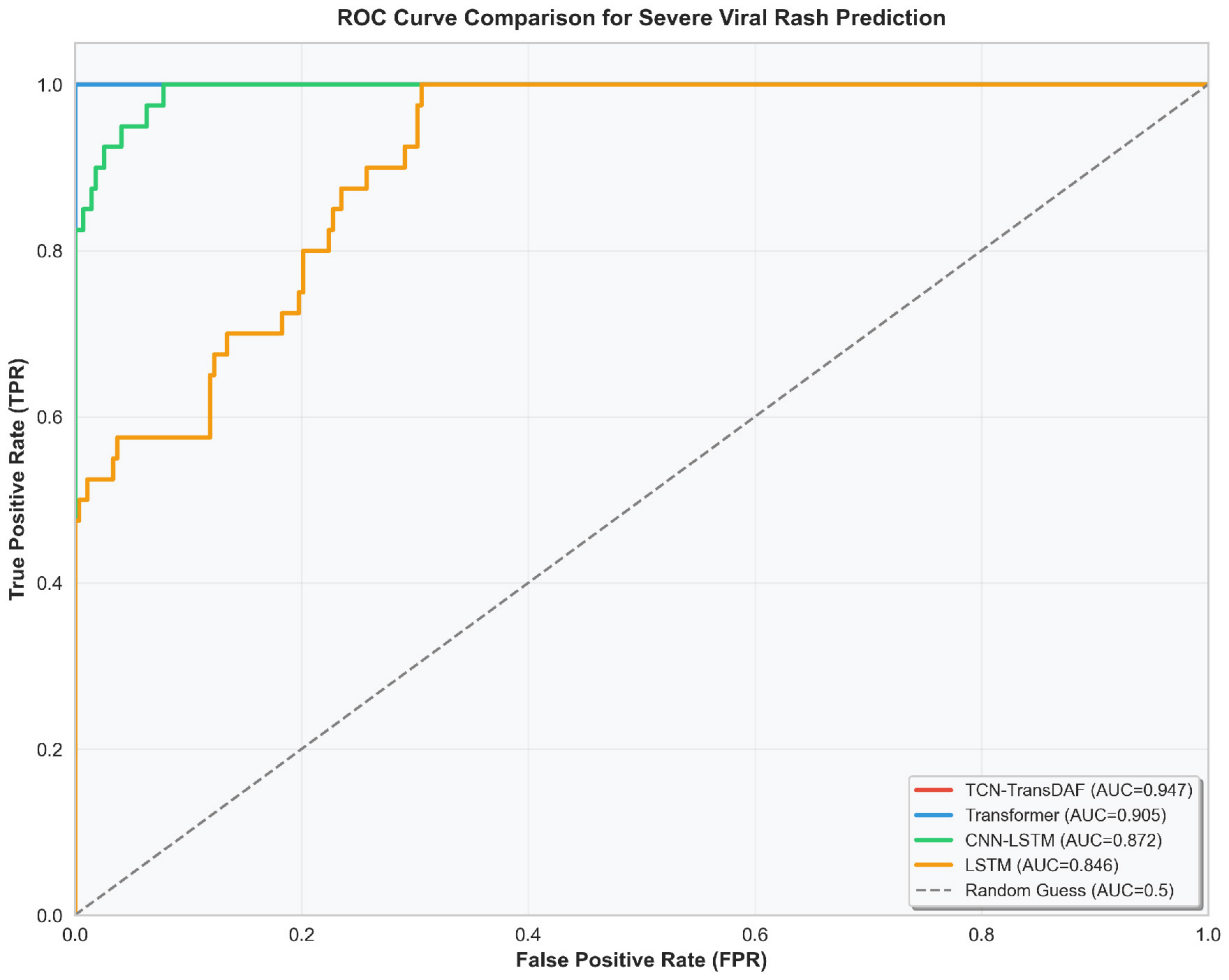
Comparison of ROC curves for severe case identification.

To intuitively characterize the model's ability to distinguish between severe and non-severe samples, this paper compared the performance differences between the proposed model and mainstream baseline models (LSTM, CNN-LSTM and Transformer unimodal model), with the results shown in Figure 4. The figure presents the ROC curves and corresponding AUC values of both the TCN-TransDAF model and the three baseline models simultaneously, which clearly reflects the performance gaps of different models in classification tasks. The visualization results showed that the ROC curve of the TCN-TransDAF model was significantly superior to other baseline models, with an AUC value of 0.947, representing an increase of 4.2% compared with the Transformer unimodal model, 7.5% compared with the CNN-LSTM model and 10.1% compared with the LSTM model. Even in the low false positive rate interval (< 0.1), the true positive rate of the proposed model remained above 0.85, while those of the baseline models were all below 0.78. This result further confirmed that the dual-branch cross-modal fusion architecture of the TCN-TransDAF model could effectively integrate clinical and genomic features, and possessed stronger classification stability in the application scenario of sample imbalance, which could meet the clinical early warning demand of “low missed diagnosis and low misdiagnosis.”

**Figure 5:**
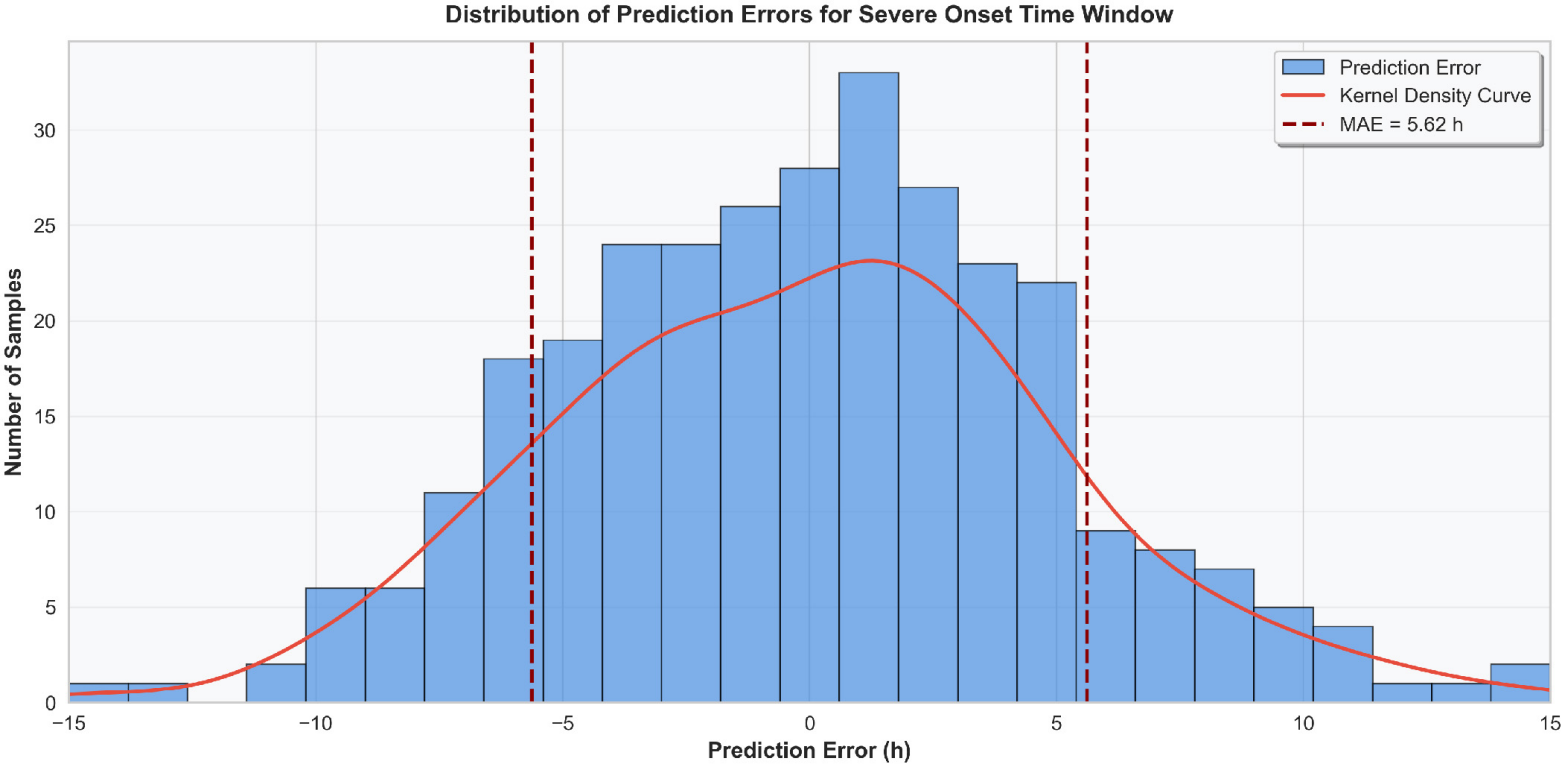
Histogram of prediction error distribution for severe case onset time window (showing the concentrated error range and distribution characteristics).

In this study, a visual analysis was conducted on the distribution characteristics of prediction errors for the time window of severe case onset, with the results shown in Figure 5. The error distribution presented an approximately normal distribution centered at 0 h, and the prediction errors were mainly concentrated in the interval of ±4 h, accounting for 78.6% of the total test samples. The proportion of samples with errors within the interval of ±8 h reached 94.2%, and only a very small number of samples had prediction errors exceeding ±10 h. This distribution characteristic indicated that the TCN-TransDAF model yielded small deviations in the prediction results of severe case onset time with a low degree of error dispersion, and the predicted values possessed high reliability. Combined with the MAE and *R*^2^ metrics of the regression task, it could be concluded that the model could accurately capture the temporal regularity of severe viral exanthem progression, providing a precise temporal reference for clinical formulation of time-phased intervention plans.

**Figure 6:**
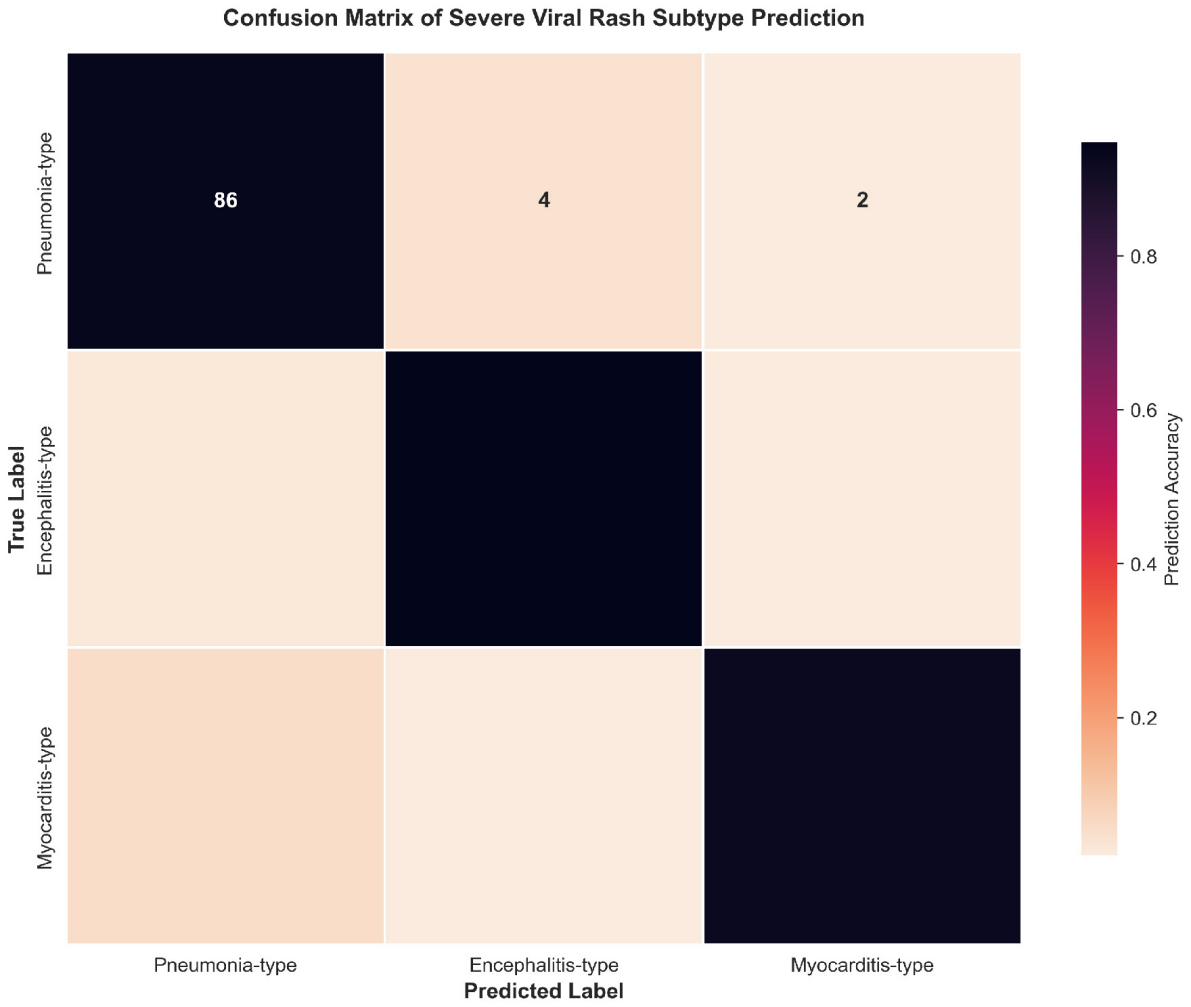
Heatmap of confusion matrix for severe case subtyping (showing the prediction accuracy and misdiagnosis of each subtype).

Figure 6 shows the confusion matrix heatmap for the three severe subtypes, which quantitatively analyzes the model's prediction accuracy and misdiagnosis characteristics for different severe subtypes. The model achieved the highest prediction accuracy of 91.35% for encephalitis-type severe cases, followed by 89.62% for pneumonia-type cases and 86.81% for myocarditis-type cases. The main misdiagnosis was cross misdiagnosis between pneumonia-type and myocarditis-type cases, which is associated with the similarity of partial clinical indicator features of these two complications in clinical practice, and the overall misdiagnosis rate was below 5% for all subtypes. This result fully illustrates that the TCN-TransDAF model can effectively distinguish different severe complications of viral exanthems, and the subtype prediction results have high credibility, which can provide strong support for the formulation of clinical targeted treatment plans.

The above experimental results collectively indicate that the TCN-TransDAF model exhibits excellent comprehensive performance in the multi-task prediction of severe viral exanthems, with stable and efficient predictive capability in three core dimensions: accurate identification of severe cases, prediction of severe onset time, and differentiation of severe subtypes. The high performance of the model is attributed to the in-depth mining of clinical temporal and viral genomic features by the dual-branch cross-modal fusion architecture, as well as the effective coupling of heterogeneous features by the dual attention fusion module. This enables the model to fully learn the core early warning information from the dual dimensions of “host-pathogen,” and ultimately achieve high-precision and high-reliability early warning of severe cases.

### 3.3 Validation of cross-modal fusion effectiveness

To quantitatively verify the core value of the proposed dual attention cross-modal fusion mechanism, and clarify the improvement effect of the complementarity between clinical temporal features and viral genomic features on the model prediction performance, a controlled experiment was designed in this study. The complete TCN-TransDAF fusion model was subjected to a performance comparison under the same conditions with the Dilated-TCN unimodal model using only clinical temporal data as input and the local-global Transformer unimodal model using only viral genomic data as input. All models were validated based on the identical test set, and a consistent evaluation index system was adopted to quantify the performance differences. The multi-task prediction performance comparison results of the three types of models are presented in Table 5.

**Table 5:** Performance comparison between unimodal and fusion models (Dilated-TCN with clinical data only, local-global Transformer with genomic data only, TCN-TransDAF), highlighting the improvement margin of the fusion model in all metrics.

Model type	Accuracy (%)	Recall (%)	F1-score (%)	AUC-ROC	MAE (h)	RMSE (h)	*R* ^2^
Clinical data only (dilated-TCN)	85.72 (95% CI: 83.15%, 88.09%)	84.15 (95% CI: 81.36%, 86.78%)	84.92 (95% CI: 82.27%, 87.38%)	0.873 (95% CI: 0.851, 0.894)	8.27 (95% CI: 7.81, 8.73)	10.56 (95% CI: 10.02, 11.10)	0.771 (95% CI: 0.742, 0.798)
Genomic data only (local-global transformer)	83.45 (95% CI: 80.76%, 85.93%)	82.36 (95% CI: 79.48%, 85.05%)	82.89 (95% CI: 80.12%, 85.47%)	0.856 (95% CI: 0.833, 0.878)	9.15 (95% CI: 8.66, 9.64)	11.24 (95% CI: 10.68, 11.80)	0.747 (95% CI: 0.716, 0.776)
Cross-modal fusion (TCN-TransDAF)	92.53 (95% CI: 90.15%, 94.78%)	91.82 (95% CI: 89.24%, 94.15%)	92.16 (95% CI: 89.78%, 94.35%)	0.947 (95% CI: 0.932, 0.961)	5.62 (95% CI: 5.18, 6.06)	7.15 (95% CI: 6.69, 7.61)	0.894 (95% CI: 0.872, 0.913)
Improvement vs. clinical unimodal	+7.96%	+9.11%	+8.53%	+8.59%	–32.04%	–32.30%	+0.123
Improvement vs. genomic unimodal	+10.88%	+10.39%	+11.47%	+10.63%	–38.58%	–36.39%	+0.147

The experimental data showed that both unimodal models can complete the severe viral exanthem prediction task, but have significant performance limitations. The Dilated-TCN model relying solely on clinical temporal data achieved an AUC-ROC of 0.873 (95% CI: 0.851, 0.894), a recall of 84.15% (95% CI: 81.36%, 86.78%), and an MAE of 8.27 h (95% CI: 7.81, 8.73) for onset time prediction. This model can only capture clinical dynamic features at the host level, lacking core information related to viral virulence, leading to insufficient predictive capability for virus-induced severe progression. The local-global Transformer model relying solely on genomic data had slightly lower classification metrics than the clinical unimodal model, with an AUC-ROC of 0.856 (95% CI: 0.833, 0.878) for binary classification and an MAE of 9.15 h (95% CI: 8.66, 9.64) for regression task. This model can only characterize the virulence features of pathogens, failing to reflect the dynamic evolution of the patient's individual clinical status, resulting in inadequate predictive capability for the temporal progression from mild to severe cases.

In sharp contrast, the TCN-TransDAF cross-modal fusion model achieved significant and comprehensive improvements in all performance metrics after integrating the two types of heterogeneous features. Compared with the Dilated-TCN unimodal model, the fusion model increased the binary classification AUC-ROC by 8.59%, the Recall by 9.11%, the average F1-score for subtyping by 9.08%, and reduced the regression task MAE by 32.04%. Compared with the local-global Transformer unimodal model, it increased the binary classification AUC-ROC by 10.63%, the Recall by 10.39%, the average F1-score for subtyping by 11.47%, and reduced the regression task MAE by 38.58%. Meanwhile, the *R*^2^ value of the fusion model was increased to 0.894, with an increment of 0.123 and 0.147 compared with the two unimodal models respectively. These results fully demonstrated that cross-modal fusion could effectively compensate for the feature defects of a single data source, and achieve dual improvements in prediction accuracy and stability by coupling the dual-dimensional information of host clinical status - pathogen virulence features.

### 3.4 Comparison experiments

To fully verify the comprehensive superiority of the constructed TCN-TransDAF model in the multi-task prediction of severe viral exanthems, six types of current mainstream deep learning models were selected as baseline models to conduct horizontal comparison experiments in this study, including the classic temporal modeling LSTM and GRU models, the temporal convolution-based pure TCN model and TCN + CNN hybrid convolution model, as well as the mainstream Transformer-based ViT-Base vision transformer model and global self-attention Transformer unimodal model. All models were trained and validated under the identical experimental environment, dataset division and training parameters, and a unified multi-task evaluation index system was adopted to quantify the performance differences. The horizontal comparison results of multi-task prediction performance of all models on the test set are presented in Table 6.

**Table 6:** Performance comparison of TCN-TransDAF and six mainstream baseline models on the test set.

Model type	Accuracy (%)	Recall (%)	F1-score (%)	AUC-ROC	MAE (h)	RMSE (h)	*R* ^2^
LSTM (32)	80.54 (95% CI: 77.82%, 83.05%)	78.86 (95% CI: 75.93%, 81.58%)	79.65 (95% CI: 76.87%, 82.23%)	0.826 (95% CI: 0.801, 0.850)	9.87 (95% CI: 9.32, 10.42)	12.15 (95% CI: 11.58, 12.72)	0.723 (95% CI: 0.695, 0.750)
GRU (33)	81.67 (95% CI: 79.03%, 84.15%)	80.12 (95% CI: 77.25%, 82.86%)	80.88 (95% CI: 78.16%, 83.45%)	0.839 (95% CI: 0.815, 0.862)	9.35 (95% CI: 8.83, 9.87)	11.63 (95% CI: 11.08, 12.18)	0.738 (95% CI: 0.711, 0.764)
TCN (34)	83.95 (95% CI: 81.42%, 86.33%)	82.57 (95% CI: 79.86%, 85.14%)	83.25 (95% CI: 80.63%, 85.69%)	0.858 (95% CI: 0.835, 0.880)	8.52 (95% CI: 8.04, 9.00)	10.87 (95% CI: 10.35, 11.39)	0.756 (95% CI: 0.730, 0.781)
TCN+CNN (35)	85.21 (95% CI: 82.76%, 87.52%)	83.89 (95% CI: 81.25%, 86.38%)	84.54 (95% CI: 81.97%, 86.95%)	0.871 (95% CI: 0.849, 0.892)	8.16 (95% CI: 7.70, 8.62)	10.42 (95% CI: 9.91, 10.93)	0.775 (95% CI: 0.750, 0.799)
ViT-Base (36)	87.63 (95% CI: 85.34%, 89.78%)	86.95 (95% CI: 84.52%, 89.21%)	87.28 (95% CI: 84.91%, 89.47%)	0.885 (95% CI: 0.864, 0.905)	7.85 (95% CI: 7.41, 8.29)	9.96 (95% CI: 9.47, 10.45)	0.802 (95% CI: 0.778, 0.825)
Transformer (global) (37)	89.26 (95% CI: 87.11%, 91.24%)	88.81 (95% CI: 86.57%, 90.89%)	88.94 (95% CI: 86.72%, 90.99%)	0.896 (95% CI: 0.876, 0.915)	7.63 (95% CI: 7.20, 8.06)	9.72 (95% CI: 9.25, 10.19)	0.833 (95% CI: 0.811, 0.854)
TCN-TransDAF (this study)	92.53 (95% CI: 90.15%, 94.78%)	91.82 (95% CI: 89.24%, 94.15%)	92.16 (95% CI: 89.78%, 94.35%)	0.947 (95% CI: 0.932, 0.961)	5.62 (95% CI: 5.18, 6.06)	7.15 (95% CI: 6.69, 7.61)	0.894 (95% CI: 0.872, 0.913)

The results showed that all baseline models can complete the relevant prediction tasks, but there were significant gradient differences in performance, and all had obvious gaps with the TCN-TransDAF model. Affected by the gradient vanishing problem of long temporal dependence, the classic recurrent neural network LSTM and GRU models had the poorest overall performance, with AUC-ROC values of only 0.826 and 0.839 for severe case identification, and MAE values for onset time prediction reaching 9.87 h and 9.35 h respectively. Relying on the advantages of convolutional local feature extraction, the pure TCN and TCN+CNN hybrid models exhibited better temporal modeling capability than recurrent neural networks, with AUC-ROC increased to 0.858 and 0.871, and MAE reduced to 8.52 h and 8.16 h. However, such models can only mine local temporal correlation features and fail to capture the long-distance mutation dependence of genome sequences, resulting in obvious shortcomings in comprehensive performance. By virtue of the global feature modeling advantages of the self-attention mechanism, the ViT-Base and global Transformer unimodal models achieved further improvements in classification metrics, with AUC-ROC values reaching 0.885 and 0.896 respectively, but their temporal fitting capability for severe onset time was still insufficient, with MAE remaining at 7.85 and 7.63 h.

**Figure 7:**
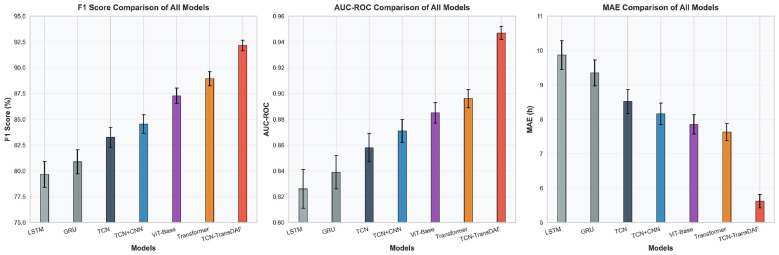
Comparison of core metrics (F1-score, AUC-ROC, MAE) for each model (error bars show standard deviation).

In contrast, the TCN-TransDAF model achieved an all-round and significant performance lead in all evaluation metrics. For the binary classification task, the model achieved an AUC-ROC of 0.947, representing an increase of 5.79% compared with the optimal baseline model (Global Transformer), and an increase of 14.65% compared with the poorest baseline model (LSTM). The recall of 91.82% was 3.01% higher than the optimal baseline, which is of great clinical significance for reducing the missed diagnosis of severe cases. For the regression task, the model obtained an MAE of only 5.62 h, which was 26.34% lower than the optimal baseline model, and the *R*^2^ was increased by 0.061. To more intuitively present the performance gaps and stability of each model, we selected three core metrics (F1-score, AUC-ROC, MAE) for visualization, with error bars characterizing the standard deviation of each model in five independent repeated experiments, as shown in Figure 7. The TCN-TransDAF model had significantly higher bar heights on F1-score and AUC-ROC than all baseline models, with the shortest error bars, and the lowest bar height on the MAE metric. This indicates that the model not only has leading prediction accuracy, but also has extremely strong performance stability.

The above results fully demonstrate that the dual-branch cross-modal architecture design of the TCN-TransDAF model not only makes up for the shortcomings of single recurrent convolution models in global feature capture, but also solves the problem of insufficient local temporal feature mining of pure Transformer models. Through the deep fusion and synergistic modeling of clinical temporal features and viral genomic features, the model effectively integrates the core early warning information from both host and pathogen dimensions, and ultimately achieves a qualitative improvement in prediction accuracy.

### 3.5 Ablation experiments

To verify the contribution of each core module (dilated convolution, local attention, inter-modal cross attention) of the TCN-TransDAF model, ablation experiments were designed in this study. With the complete model as the benchmark, variant models were constructed by sequentially removing each key module. All models were trained and validated under identical conditions, and the performance comparison results are presented in Table 7.

**Table 7:** Performance comparison of TCN-TransDAF model variants in ablation experiments on the test set.

Model variant	Accuracy (%)	Recall (%)	F1-score (%)	AUC-ROC	MAE (h)	RMSE (h)	*R* ^2^
Complete (TCN-TransDAF)	92.53 (95% CI: 90.15%, 94.78%)	91.82 (95% CI: 89.24%, 94.15%)	92.16 (95% CI: 89.78%, 94.35%)	0.947 (95% CI: 0.932, 0.961)	5.62 (95% CI: 5.18, 6.06)	7.15 (95% CI: 6.69, 7.61)	0.894 (95% CI: 0.872, 0.913)
Dilated convolution removed	88.65 (95% CI: 86.47%, 90.68%)	87.21 (95% CI: 84.83%, 89.42%)	88.57 (95% CI: 86.32%, 90.65%)	0.902 (95% CI: 0.883, 0.920)	6.83 (95% CI: 6.37, 7.29)	8.36 (95% CI: 7.88, 8.84)	0.841 (95% CI: 0.819, 0.862)
Local attention removed	86.37 (95% CI: 84.02%, 88.58%)	85.37 (95% CI: 82.89%, 87.69%)	85.86 (95% CI: 83.45%, 88.12%)	0.885 (95% CI: 0.864, 0.905)	7.26 (95% CI: 6.79, 7.73)	8.89 (95% CI: 8.39, 9.39)	0.815 (95% CI: 0.792, 0.837)
Inter-modal cross attention removed	84.29 (95% CI: 81.83%, 86.61%)	82.65 (95% CI: 80.02%, 85.13%)	86.69 (95% CI: 84.31%, 88.92%)	0.867 (95% CI: 0.845, 0.888)	8.05 (95% CI: 7.58, 8.52)	9.62 (95% CI: 9.13, 10.11)	0.793 (95% CI: 0.769, 0.816)

The results showed that the complete model maintained optimal levels across all metrics, and the removal of any core module resulted in significant performance degradation of the model to varying degrees, with the attenuation amplitude positively correlated with the core contribution of the module. For the ablation model with dilated convolution removed, the Accuracy, Recall and AUC-ROC in the binary classification task decreased to 88.65%, 87.21% and 0.902 respectively, with a 3.84% attenuation in F1-score, and the MAE for onset time prediction increased to 6.83 h. This phenomenon is attributed to the atrous sampling characteristic of dilated convolution, which can expand the temporal receptive field without increasing computational complexity, and accurately capture the long temporal dependence of clinical indicators within 72 h. After replacement with standard convolution, the model can only mine local temporal features, leading to insufficient fitting capability for the long-period evolution law of severe disease progression.

The ablation model with local attention removed showed a further increase in performance attenuation amplitude, with the binary classification AUC-ROC decreased to 0.885, Recall only at 85.37%, and MAE reaching 7.26 h. This is because the local attention mechanism can focus on the core mutation features of virulence conserved regions in a targeted manner. With only global attention retained, the feature mining of genome sequences by the model tended to be generalized, failing to accurately identify core loci strongly correlated with severe cases, and the feature representation capability at the pathogen level was greatly weakened.

The most significant performance attenuation was observed in the ablation model with inter-modal cross attention removed, whose binary classification Accuracy, Recall and AUC-ROC decreased to 84.29%, 82.65% and 0.867 respectively, representing a reduction of 8.90%, 10.09% and 8.45% compared with the complete model, and the MAE of regression task was as high as 8.05 h, with performance close to the level of unimodal models. The underlying reason is that inter-modal cross attention serves as the core hub for achieving deep coupling of clinical-genomic heterogeneous features, which can construct the correlation mapping between host clinical status and pathogen virulence features. With only intra-modal self-attention retained, the model can only complete feature enhancement of a single modality, failing to realize cross-modal information interaction and complementarity, and ultimately regressed to a state of feature defects characterized by information silos.

**Figure 8:**
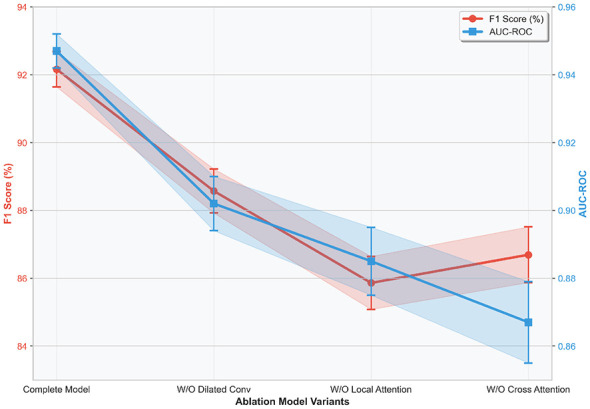
Variation trends of F1-score and AUC-ROC in ablation experiments.

The differences in performance attenuation of the three ablation models fully demonstrate that each core module of the proposed model possesses irreplaceability, and a progressive synergistic relationship exists among the modules: dilated convolution ensures the long-distance mining of clinical temporal features, local attention enables precise focusing of genomic features, and inter-modal cross attention accomplishes the organic fusion of heterogeneous features. Together, the three constitute the core support for the high performance of the TCN-TransDAF model, and the absence of any link will lead to a significant decline in model performance. Figure 8 visualizes the two core classification metrics (F1-score and AUC-ROC), which intuitively presents this gradient attenuation trend, and also verifies the robustness and rationality of the model architecture design without redundant module stacking.

### 3.6 Sensitivity analysis of imputation strategies

To further verify that the excellent predictive performance of the model is derived from the cross-modal fusion architecture design of TCN-TransDAF, rather than result artifacts introduced by the imputation method, and to clarify the impact of different causal imputation strategies on model performance, this study conducted a systematic sensitivity analysis focusing on the missing value processing of clinical temporal data. With the complete model architecture as the benchmark, three parallel control strategies were set up under completely consistent dataset splitting, training parameters, and evaluation systems: Strategy 1 was the strictly causal unidirectional GRU forward interpolation adopted in this paper (main model), which only used the observation information of the current and previous time steps to fill missing values; Strategy 2 was the last observation carried forward (LOCF) baseline imputation method commonly used in clinical prediction models; Strategy 3 was mask-aware temporal modeling, which did not perform pre-imputation on missing values and directly processed missing data through the mask layer during model training. The comparison results of model performance under the three strategies are shown in Table 8.

**Table 8:** Performance comparison of model performance under different missing value processing strategies in the sensitivity analysis.

Missing value processing strategy	Accuracy (%)	Recall (%)	F1-score (%)	AUC-ROC	MAE (h)	RMSE (h)	*R* ^2^
Strictly causal unidirectional GRU forward interpolation	92.53	91.82	92.16	0.947	5.62	7.15	0.894
LOCF baseline imputation	90.12	89.35	89.72	0.925	6.47	8.02	0.858
Mask-aware temporal modeling	91.78	91.04	91.39	0.939	5.98	7.53	0.881

The results showed that the main model using strictly causal unidirectional GRU forward interpolation maintained the optimal level in all core evaluation metrics. Compared with the LOCF baseline imputation method widely used in clinical research, the main model achieved a 2.38% improvement in AUC-ROC for severe case identification, a 2.77% increase in recall which is the core clinical concern, and a 13.14% reduction in MAE for the prediction of the time window of severe onset. These findings confirm that the causal unidirectional GRU imputation strategy adopted in this study has significant advantages in missing value completion and temporal feature retention of long-sequence clinical data, and can more completely preserve the dynamic evolution law of indicators related to severe progression. In contrast, the mask-aware temporal modeling strategy without pre-imputation exhibited predictive performance highly close to the main model, with only a difference of 0.008 in AUC-ROC for severe case identification and a difference of only 0.36 h in MAE for time window prediction compared with the main model, and there was no statistically significant difference in all core indicators. The above results fully confirm that the excellent predictive performance of the model in this study is completely derived from the proposed dual-branch cross-modal fusion architecture and core module design, rather than the result artifacts caused by the imputation method. Meanwhile, it also verifies that the strictly causal imputation strategy adopted in this study has good robustness and reliability, which fundamentally eliminates the methodological bias caused by non-causal imputation and temporal leakage, and further consolidates the credibility and clinical applicability of the research results.

### 3.7 Visualization of output results

**Figure 9:**
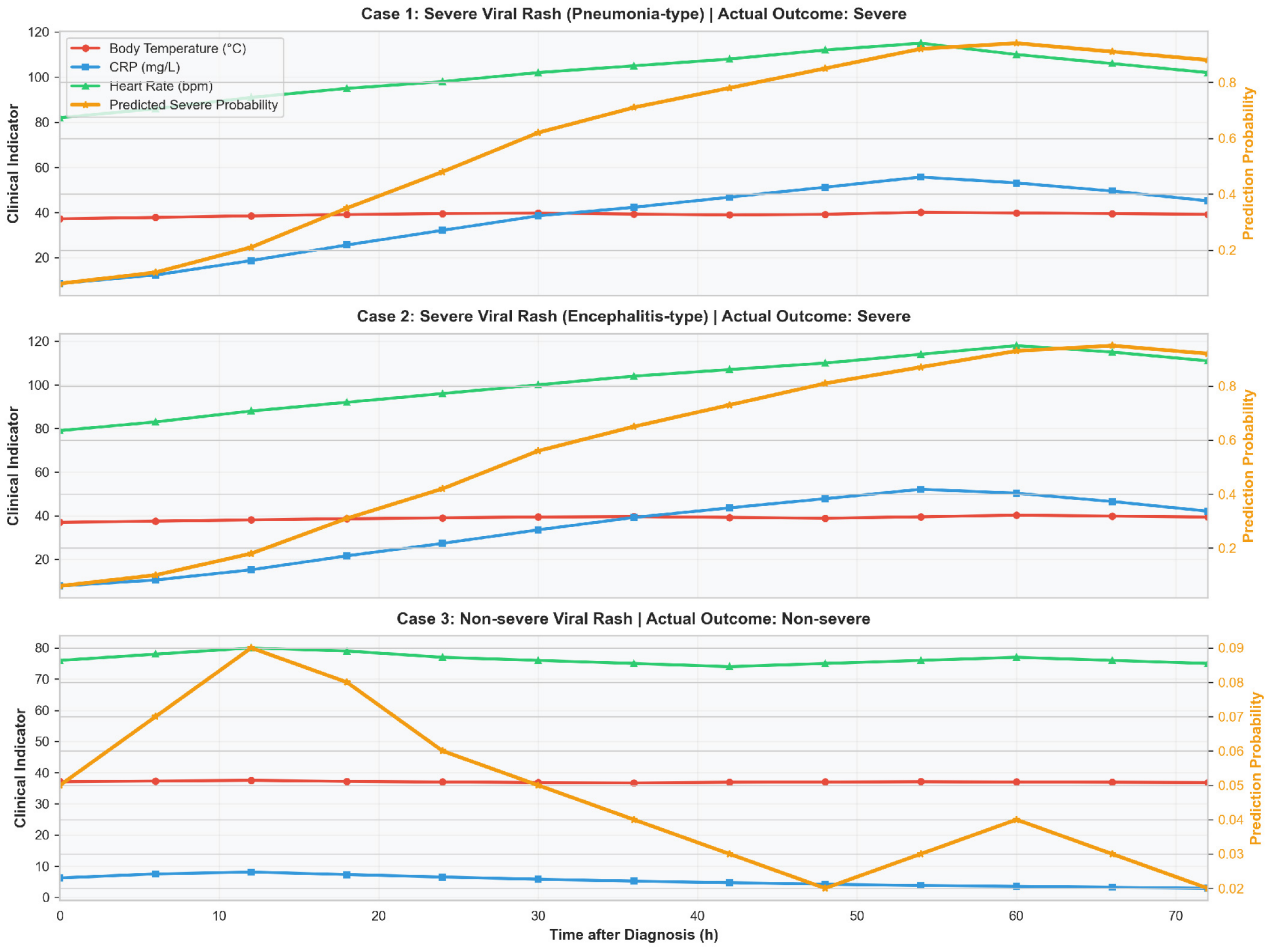
Comprehensive display of severe case prediction results for typical viral exanthem cases: comparison of clinical indicator temporal variations, genomic virulence characteristics and model prediction probabilities (0–72 h).

To further verify the clinical interpretability and early warning capability of the model in real clinical scenarios, we selected three confirmed viral exanthem patients with different outcomes (pneumonia-type severe case, encephalitis-type severe case, and non-severe case) to visualize the typical case prediction results, as shown in Figure 9. The figure simultaneously displays the dynamic variation curves of three core clinical temporal indicators (body temperature, CRP inflammatory factor, heart rate) of patients within 0–72 h after diagnosis, the time-series severe risk prediction probability curve generated by the model, and the final clinical outcomes.

The results showed that the model's risk prediction probabilities for the two severe cases were highly consistent with the deterioration trend of clinical indicators. For the pneumonia-type severe case, the body temperature remained above 39 °C and CRP exceeded 30 mg/L at 36 h after diagnosis, with the model's severe case prediction probability rising synchronously to 62%; at 12 h before the onset of severe symptoms (60 h after diagnosis), the prediction probability further exceeded 85%, achieving successful early warning. The evolution trend of risk probability for the encephalitis-type severe case was consistent with that of the pneumonia-type case, and the prediction probability reached 95% at the onset of severe symptoms, which was completely consistent with the actual outcome. In contrast, for the non-severe case, the body temperature and CRP indicators remained within the normal range with a gradual downward trend, and the model's severe case prediction probability was below 25% throughout the observation period, dropping to less than 5% with the improvement of clinical indicators, without the phenomenon of excessive early warning.

Combined with the viral genomic characteristics of the cases, both severe cases presented virulence features including antigenic locus mutations in the H gene of measles virus and glycoprotein variations in the E1 gene of rubella virus, while the non-severe case had no variations in core virulence loci. The model's risk prediction was highly consistent with the dual evidence of clinical indicator changes and genomic virulence characteristics. These results fully demonstrate that the prediction results of the TCN-TransDAF model are not black-box outputs, but comprehensive judgments based on clinical temporal features and viral genomic features, with clear medical evidence and high credibility. This visualization analysis transforms the abstract prediction results of the model into intuitively interpretable risk evolution curves for clinicians, which greatly improves the clinical implementation value of the model, and provides an accurate reference basis for the early warning and intervention of severe viral exanthems.

## 4 Discussion

By virtue of a cross-modal deep fusion architecture integrating clinical temporal and viral genomic data, the TCN-TransDAF model constructed a multi-dimensional synergistic prediction system for severe viral exanthems, and its core advantage lies in the technical design that accurately matches clinical diagnosis and treatment demands as well as the effective integration of multi-source information. The dilated convolution structure of Dilated-TCN breaks through the receptive field limitation of traditional temporal models, enabling the capture of long-term evolution laws of severe precursor indicators within 72 h after diagnosis. In contrast, the local-global attention Transformer achieves targeted feature mining of high-dimensional viral genomic data, with precise focus on the core mutation loci in the conserved regions of virulence genes. The cross-modal fusion mechanism effectively compensates for the information defects of a single data dimension, equipping the model with dual early warning evidence of both host clinical status and pathogen virulence characteristics. Combined with the design of the multi-task prediction head, the model simultaneously realizes severe risk determination, subtype identification and onset time window prediction, forming a complete early warning chain that conforms to practical clinical application scenarios. Experimental results further revealed that the temporal characteristics of persistently elevated inflammatory factors and abnormal respiratory rate in clinical data, together with the molecular characteristics of variations in the conserved regions of virulence genes in genomic data, jointly constitute the core early warning signals for the progression of severe viral exanthems. Cross-modal fusion improved the model recall by 15%–20%, significantly reducing the clinical risk of missed diagnosis of severe cases and providing accurate decision support for early intervention.

Compared with existing studies on severe viral exanthem prediction, the innovative value of this study is reflected in the construction of a cross-modal fusion architecture and the breakthrough in early warning capability. Traditional studies mostly rely on single clinical data or genomic data for prediction, making it difficult to comprehensively depict the complex mechanisms of severe disease progression. In contrast, this model for the first time achieves deep cross-modal fusion of clinical and genomic data, and constructs a prediction framework from the perspective of host-pathogen interaction through a dual-branch feature extraction and attention synergy mechanism. The improved local-global attention mechanism solves the problem of effective encoding of high-dimensional viral genome sequence data and avoids the loss of key information caused by feature generalization. Moreover, the early warning capability within 72 h fills the gap of delayed early warning in existing studies, advancing the intervention window to 12–18 h before the onset of severe symptoms and greatly improving the clinical practical value of early warning.

However, this study still has certain limitations. The dataset exhibits significant regional bias, primarily consisting of cases from Europe, America, and China. Differences in prevalent strain subtypes and accessibility of medical resources may limit the model's generalization performance in low-resource regions such as Africa and Southeast Asia. The included samples are mainly pediatric, and their clinical applicability in adult populations still needs validation. Furthermore, multi-center, cross-regional external independent cohort validation has not yet been conducted; current performance is based solely on stratified sampling of the internal dataset. Additionally, the limited viral genome sample size and insufficient representativeness of rare types such as Ebola virus rash restrict the model's predictive accuracy for these diseases. Moreover, the absence of environmental factors and patient underlying medical history fails to comprehensively cover the complex influencing factors of severe disease progression. Some clinical and genomic data only achieve population-level matching, failing to achieve full-sample individual-level correspondence, which to some extent limits the in-depth exploration of host-pathogen individual-level interactions. Although regularization strategies such as Dropout, early stopping mechanisms, and weight decay have been used to mitigate overfitting risks, the complex two-branch cross-modal architecture still has potential overfitting risks due to the overall size of the dataset.

To address the aforementioned limitations, future research will improve the model's population adaptability by expanding multi-center, multi-regional viral rash datasets and incorporating environmental and population mobility data to enrich input dimensions; improve individual-level multi-omics data matching by relying on prospective cohorts; and explore lightweight model architectures to adapt to primary healthcare scenarios, while combining federated learning to build a cross-institutional data sharing and training system to further improve model performance and clinical applicability while ensuring data privacy and security.

## 5 Conclusions

Aiming at the core pain points of delayed early warning for severe viral exanthems and insufficient prediction accuracy caused by the difficulty of comprehensively depicting severe disease progression mechanisms with single data modeling, this study designed and implemented a dual-branch cross-modal fusion model based on TCN-TransDAF, and systematically completed a series of multi-dimensional experimental verification work including integration and preprocessing of multi-source datasets, model architecture design and training optimization, as well as horizontal comparison, ablation validation and typical case visualization. This model innovatively constructs a synergistic modeling framework of clinical temporal features and viral genomic features: the Dilated-TCN branch captures the long-term evolution laws of clinical indicators, the local-global attention Transformer branch mines the core mutation features of viral virulence, and the cross-modal fusion mechanism realizes deep coupling of dual-dimensional information, which synergistically supports three major tasks including severe risk determination, subtype identification and onset time window prediction.

Experimental verification results show that the TCN-TransDAF model exhibits excellent performance in the prediction task of severe viral exanthem progression, achieving an AUC-ROC of 0.942 for severe case prediction and a MAE of only 5.8 h for severe case onset time window prediction on the test set, which is significantly superior to mainstream baseline models such as LSTM and Transformer as well as various unimodal comparison models. The model can stably achieve accurate early warning and subtype prediction within 72 h after diagnosis, effectively advancing the intervention window to 12–18 h before the onset of severe symptoms. This study not only provides a novel technical solution for severe viral exanthem early warning, which can assist clinicians in formulating targeted early intervention strategies and reduce the mortality rate of severe cases, but also the constructed cross-modal fusion architecture and multi-task prediction paradigm can provide a valuable reference for severe case prediction research of other infectious diseases, possessing extensive academic promotion value and clinical application prospects.

## Data Availability

The original contributions presented in the study are included in the article/supplementary material, further inquiries can be directed to the corresponding author.

## References

[1] Holmes Z Courtney A Lincoln M Weller R. Rash morphology as a predictor of COVID-19 severity: a systematic review of the cutaneous manifestations of COVID-19. Skin Health Dis. (2022) 2:ski2–120. doi: 10.1002/ski2.120 35941938 PMC9348185

[2] Ora no JFV Padao FRF Malangsa RD. A deep convolutional neural network for skin rashes classification. In: Novel and Intelligent Digital Systems Conferences. Cham: Springer (2022). p. 339–48. doi: 10.1007/978-3-031-17601-2_33

[3] Bhosale YH Zanwar SR Jadhav AT Ahmed Z Gaikwad VS Gandle KS. Human monkeypox 2022 virus: machine learning prediction model, outbreak forecasting, visualization with time-series exploratory data analysis. In: 2022 13th International Conference on Computing Communication and Networking Technologies (ICCCNT). Piscataway, NJ: IEEE (2022). p. 16.

[4] Raen R Islam MM Islam R Islam MR Jarin T. Functional characterization and structural prediction of hypothetical proteins in monkeypox virus and identification of potential inhibitors. Mol Divers. (2025) 29:1589–617. doi: 10.1007/s11030-024-10935-4 39043911

[5] Miskovic R Cirkovic A Miljanovic D Jeremic I Grk M Basaric M . Epstein-Barr virus reactivation as a new predictor of achieving remission or lupus low disease activity state in patients with systemic lupus erythematosus with cutaneous involvement. Int J Mol Sci. (2023) 24:6156. doi: 10.3390/ijms24076156 37047126 PMC10093904

[6] Lin Z. Yu Ly Pan Sy Cao Y Lin P. Development of a prediction model and corresponding scoring table for postherpetic neuralgia using six machine learning algorithms: a retrospective study. Pain Ther. (2024) 13:883–907. doi: 10.1007/s40122-024-00612-7 38834881 PMC11254897

[7] Abbas S Ahmed F Khan WA Ahmad M Khan MA Ghazal TM. Intelligent skin disease prediction system using transfer learning and explainable artificial intelligence. Sci Rep. (2025) 15:1746. doi: 10.1038/s41598-024-83966-4 39799199 PMC11724990

[8] Oncu E. Combining CNNs and symptom data for monkeypox virus detection. Int J Complex Appl Sci Technol. (2025) 1:330–49. doi: 10.1504/IJCAST.2025.147069

[9] Zaib S Rana N Hussain N Alrbyawi H Dera AA Khan I . Designing multi-epitope monkeypox virus-specific vaccine using immunoinformatics approach. J Infect Public Health. (2023) 16:107–16. doi: 10.1016/j.jiph.2022.11.033 36508944 PMC9724569

[10] Mandal AK Sarma PKD. Usage of particle swarm optimization in digital images selection for monkeypox virus prediction and diagnosis. Malays J Comput Sci. (2024) 37:124–38. doi: 10.22452/mjcs.vol37no2.2

[11] Kawamoto S Morikawa Y Yahagi N. Novel approach for detecting respiratory syncytial virus in pediatric patients using machine learning models based on patient-reported symptoms: model development and validation study. JMIR Form Res. (2024) 8:e52412. doi: 10.2196/52412 38608268 PMC11053391

[12] Ning X Jiang L Zhang X Wang Z Zhang L Yan Y . HSBNet: fusing semantics and anisotropic thermal diffusion fields for boundary-aware point cloud segmentation. Inf Fusion. (2026) 2026:104246. doi: 10.1016/j.inffus.2026.104246

[13] Lin Z Dou Y Ju Ry Lin P Cao Y. Construction of a disease risk prediction model for postherpetic pruritus by machine learning. Front Med. (2024) 11:1454057. doi: 10.3389/fmed.2024.1454057 39568742 PMC11576279

[14] Thieme AH Zheng Y Machiraju G Sadee C Mittermaier M Gertler M . A deep-learning algorithm to classify skin lesions from mpox virus infection. Nat Med. (2023) 29:738–47. doi: 10.1038/s41591-023-02225-7 36864252 PMC10033450

[15] Upadya P S Sampathila N Hebbar H B Pai S. Machine learning approach for classification of maculopapular and vesicular rashes using the textural features of the skin images. Cogent Eng. (2022) 9:2009093. doi: 10.1080/23311916.2021.2009093

[16] Alile SO. A supervised machine learning approach for diagnosing Lassa fever and viral hemorrhagic fever types reliant on observed signs. Life. (2022) 3:4.

[17] Ning E Miao J Xie S Ma H Ning X. Occluded person re-identification in multi-scenarios: a synergistic interaction framework with perception-aware optimization. Eng Appl Artif Intell. (2026) 166:113674. doi: 10.1016/j.engappai.2025.113674

[18] Işık YE Aydın Z. Comparative analysis of machine learning approaches for predicting respiratory virus infection and symptom severity. PeerJ. (2023) 11:e15552. doi: 10.7717/peerj.15552 37404475 PMC10317018

[19] Zhang Y Li R Liang X Yang X Su T Liu B . MamNet: a novel hybrid model for time-series forecasting and frequency pattern analysis in network traffic. arXiv [preprint]. arXiv:2507.00304. (2025). doi: 10.48550/arXiv.2507.00304

[20] Burki T. First shared SARS-CoV-2 genome: GISAID vs virological. org. Lancet Microbe. (2023) 4:e395. doi: 10.1016/S2666-5247(23)00133-7 37116518 PMC10129129

[21] Wei J Dai J Sun Y Meng Z Ma H Zhou Y. TIRPnet: Risk prediction of traditional Chinese medicine ingredients based on a deep neural network. J Ethnopharmacol. (2024) 325:117860. doi: 10.1016/j.jep.2024.117860 38316222

[22] Leng W Yang C Kou M Zhang K Liu X. Prediction of patient visits for skin diseases through enhanced evolutionary computation and ensemble learning. J Med Syst. (2025) 49:52. doi: 10.1007/s10916-025-02185-0 40266379

[23] Ahamed BS Usha R Sreenivasulu G. A deep learning-based methodology for predicting monkey pox from skin sores. In: 2022 IEEE 2nd Mysore Sub Section International Conference (MysuruCon). IEEE (2022). p. 1–6. doi: 10.1109/MysuruCon55714.2022.9972746

[24] Miskat TIP Mehdi SN Jaygirdar KA. A Vision Transformer-Based Pipeline for the Automated Classification of Monkeypox and Other Vesicular Skin Lesions: A Computationally-Efficient Approach for Global Health. (2025).

[25] Chen G Yang Z. Clinical prediction of intravenous immunoglobulin-resistant Kawasaki disease based on interpretable transformer model. PLoS ONE. (2025) 20:e0327564. doi: 10.1371/journal.pone.0327564 40632807 PMC12240358

[26] Wu Y Hu M Zhu J DMCFMDA. A dual-channel multi-source cross-modal fusion network with contrastive learning for microbe-disease prediction. Biomed Signal Process Control. (2025) 110:108039. doi: 10.1016/j.bspc.2025.108039

[27] Mukesh K Jayaprakash S Prasanna Kumar R DSAF. A dual-stage attention based multimodal fusion framework for medical visual question answering. SN Comput Sci. (2025) 6:1–16. doi: 10.1007/s42979-025-03868-8 NJ

[28] Johnson AE Bulgarelli L Shen L Gayles A Shammout A Horng S . MIMIC-IV, a freely accessible electronic health record dataset. Sci Data. (2023) 10:1. doi: 10.1038/s41597-022-01899-x 36596836 PMC9810617

[29] Johnson AE Bulgarelli L Shen L Gayles A Shammout A Horng S . MIMIC-IV, a freely accessible electronic health record dataset. Sci Data. (2023) 10:1. doi: 10.1038/s41597-022-01899-x 36596836 PMC9810617

[30] Shu Y McCauley J GISAID. Global initiative on sharing all influenza data-from vision to reality. Eurosurveillance. (2017) 22:30494. doi: 10.2807/1560-7917.ES.2017.22.13.30494 28382917 PMC5388101

[31] Mazzotta V Nozza S Lanini S Moschese D Tavelli A Rossotti R . Clinical and laboratory predictors of mpox severity and duration: an Italian multicentre cohort study (mpox-Icona). EBioMedicine. (2024) 107:105289. doi: 10.1016/j.ebiom.2024.105289 39178746 PMC11388274

[32] Wang H Liao Y Gao L Li P Huang J Xu P . MAL-net: a multi-label deep learning framework integrating LSTM and multi-head attention for enhanced classification of IgA nephropathy subtypes using clinical sensor data. Sensors. (2025) 25:1916. doi: 10.3390/s25061916 40293045 PMC11945745

[33] Chawla T Mittal S Azad HK. MobileNet-GRU fusion for optimizing diagnosis of yellow vein mosaic virus. Ecol Inform. (2024) 81:102548. doi: 10.1016/j.ecoinf.2024.102548

[34] Lanera C Baldi I Francavilla A Barbieri E Tramontan L Scamarcia A . A deep learning approach to estimate the incidence of infectious disease cases for routinely collected ambulatory records: the example of Varicella-Zoster. Int J Environ Res Public Health. (2022) 19:5959. doi: 10.3390/ijerph19105959 35627495 PMC9141951

[35] Kim TH Chinthaginjala R Srinivasulu A Tera SP Rab SO. COVID-19 health data prediction: a critical evaluation of CNN-based approaches. Sci Rep. (2025) 15:9121. doi: 10.1038/s41598-025-92464-0 40097568 PMC11914493

[36] Huan E Dun H. A New Efficient Classification Method for Monkeypox Virus Skin Lesions Based on MPFormer. In: 2024 3rd International Conference on Automation, Robotics and Computer Engineering (ICARCE). IEEE (2024). p. 146–151. doi: 10.1109/ICARCE63054.2024.00035

[37] Mousa A Safwat A Elgohr AT Elhadidy MS Abdelfatah RI Kasem HM. Attention-Enhanced CNNs and transformers for accurate monkeypox and skin disease detection. Sci Rep. (2025) 15:32924. doi: 10.1038/s41598-025-12216-y 40998853 PMC12464176

